# Recent Developments in 3D-(Bio)printed Hydrogels as Wound Dressings

**DOI:** 10.3390/gels10020147

**Published:** 2024-02-14

**Authors:** Olga Kammona, Evgenia Tsanaktsidou, Costas Kiparissides

**Affiliations:** 1 Chemical Process & Energy Resources Research Institute, Centre for Research and Technology Hellas, P.O. Box 60361, 57001 Thessaloniki, Greece; jtsanaktsidou@certh.gr (E.T.); costas.kiparissides@certh.gr (C.K.); 2 Department of Chemical Engineering, Aristotle University of Thessaloniki, P.O. Box 472, 54124 Thessaloniki, Greece

**Keywords:** hydrogels, wound healing, 3D bioprinting, wound dressings, polymers

## Abstract

Wound healing is a physiological process occurring after the onset of a skin lesion aiming to reconstruct the dermal barrier between the external environment and the body. Depending on the nature and duration of the healing process, wounds are classified as acute (e.g., trauma, surgical wounds) and chronic (e.g., diabetic ulcers) wounds. The latter take several months to heal or do not heal (non-healing chronic wounds), are usually prone to microbial infection and represent an important source of morbidity since they affect millions of people worldwide. Typical wound treatments comprise surgical (e.g., debridement, skin grafts/flaps) and non-surgical (e.g., topical formulations, wound dressings) methods. Modern experimental approaches include among others three dimensional (3D)-(bio)printed wound dressings. The present paper reviews recently developed 3D (bio)printed hydrogels for wound healing applications, especially focusing on the results of their in vitro and in vivo assessment. The advanced hydrogel constructs were printed using different types of bioinks (e.g., natural and/or synthetic polymers and their mixtures with biological materials) and printing methods (e.g., extrusion, digital light processing, coaxial microfluidic bioprinting, etc.) and incorporated various bioactive agents (e.g., growth factors, antibiotics, antibacterial agents, nanoparticles, etc.) and/or cells (e.g., dermal fibroblasts, keratinocytes, mesenchymal stem cells, endothelial cells, etc.).

## 1. Introduction

Skin is considered the first line of the body’s defense against external invaders causing various diseases since it protects the internal organs/tissues from direct sunlight (i.e., UV radiation), extreme temperatures, injuries, infections, etc. Hence, it is a very vulnerable tissue highly prone to injury, resulting in disruption of epidermal and probably dermal integrity or otherwise in skin wounds [1,2,3,4]. Wounds can be classified as acute and chronic depending on the healing process’s nature and duration. Acute wounds such as trauma and surgical wounds usually heal in a relatively short time frame (e.g., 2–3 months) depending on the epidermal/dermal damage, size and depth of the wound. On the other hand, chronic wounds such as pressure, venous and diabetic ulcers do not heal (non-healing chronic wounds) or take a very long time to heal (e.g., several months), resulting in the formation of scars, and can be infected by bacteria and other exogenous factors [2,5]. Chronic wounds affect millions of people (≥20 million) and represent a significant source of morbidity and an important financial burden to healthcare systems and society in general, consisting of direct (e.g., medical, health care) and indirect (e.g., sick leave, loss in productivity, early retirement, etc.) costs which increase year after year. This is mainly due to the aging population (i.e., chronic wounds are mostly common in elderly people [4,6]) and the modern lifestyle involving increased food consumption and lack of exercise [1,5,7,8]. For example, the global prevalence of obesity strongly increases the risk of diabetes and the development of diabetic foot ulcers, which have a serious impact on quality of life, due to the extremely slow healing process, recurrent infection (in ≥50% of diabetic ulcers), and in worst cases amputation (~20% of diabetic ulcer patients) [9] and mortality [8]. Even though there exist various treatments for diabetic wounds (e.g., surgical debridement, cell and/or growth factor delivery, vascular reconstruction, etc.), they usually have an enormous cost and limited efficiency [9]. Accordingly, the healing of chronic wounds is considered a major clinical challenge.

Wound dressings are sterile pads typically applied to wounds to stop bleeding, absorb exudate and protect the wounded area from being infected by bacteria and potentially other microorganisms, as well as from further trauma, thus promoting wound healing [5]. Medical adhesives such as fibrin glue, polyethylene glycol (PEG)- and cyanoacrylate-based adhesives have been extensively used for wound management and have been found to promote wound healing. However, difficulties in removing these adhesives due to strong attachment to the newly grown granulation tissue resulting in further injury and infection, limited oxygen permeability, inability for sufficient drug loading and absence of biomimicry have led to the development and exploration of advanced multifunctional wound dressings (e.g., patches with micro-, nano-architecture). The latter are capable of providing protection, maintaining the moisture in the wound microenvironment and accelerating the healing rate. Appropriate dressings should be selected as medical treatment for specific wounds taking into account the type of wound, its depth, its anatomical location, etc. [8,10]. The novel multifunctional wound dressings aim to address the great medical need to heal chronic wounds. Current treatment methods include skin flaps, full-thickness grafts, dermal substitutes, etc., which, however, suffer from donor sites’ shortage and often result in the formation of hypertrophic scars [8]. Tissue engineering (TE) approaches have led to the development of various temporary and permanent dressings that still do not meet the mechanical strength and skin biomimicry requirements [8].

Hydrogels have received a lot of attention as possible wound dressings owing to their potential to mimic characteristics of the extracellular matrix (ECM) environment present in native tissues [11,12], their elasticity (which plays a critical role in engineering soft and elastic tissues such as skin) and their high water content, which creates a cooling effect and facilitates the dressing application and removal, as well as their ability to encapsulate various bioactives (e.g., growth factors, antimicrobials, etc.) [2,5]. Recent advances in 3D (bio)printing technologies permit hydrogel customization according to wound size and depth [13,14] and enable the formation of multi-component hydrogels exhibiting various microstructures and networks of interconnected pores, which facilitate the transport of oxygen, nutrients, metabolic wastes, etc. [13,14]. Additionally, 3D bioprinting allows the temporal and spatial control of bioactive release, thus promoting bacteria reduction, favoring tissue proliferation and decreasing healing time [14]. An important advantage of hydrogel bioprinting, in comparison with traditional fabrication methods, is that it enables accurate control over the spatial patterning and architecture of constructs containing cellular and biochemical components, thus allowing the replication of biological tissues’ native spatial organization [15].

The aim of the present work is to comprehensively review the various types of 3D-(bio)printed hydrogel constructs (e.g., bioactive/antibacterial hydrogels, composite hydrogels, cell-laden hydrogels) that have been used in wound healing applications. The constructs are methodically presented in a tabulated form giving detailed information concerning the selected materials (e.g., sodium alginate, gelatin, peptides, etc.), the crosslinking (e.g., ionic, chemical) and (bio)printing methods (e.g., extrusion, digital light processing, etc.), the type of encapsulated bioactives (e.g., growth factors, antibacterial agents, etc.), the type of printed cells (e.g., fibroblasts, keratinocytes, stem cells, blood cells, etc.), the animal model used (e.g., normal and diabetic mice, rats, rabbits, etc.) and the type of wound (e.g., full-thickness wound), as well as the research outcome regarding physicochemical characterization of the (bio)printed hydrogel constructs, and in vitro and in vivo testing.

The present review paper is based on methodical research from PubMed and Google Scholar, using a combination of the following search terms: hydrogels, wound healing and 3D printing. The research covered a time period from 1 January 2013 to today. Both research and review papers addressing the in vitro and/or in vivo examination of various types of 3D-(bio)printed hydrogels for wound healing applications were carefully evaluated and chosen for inclusion.

## 2. Wound Healing

**Figure 1:**
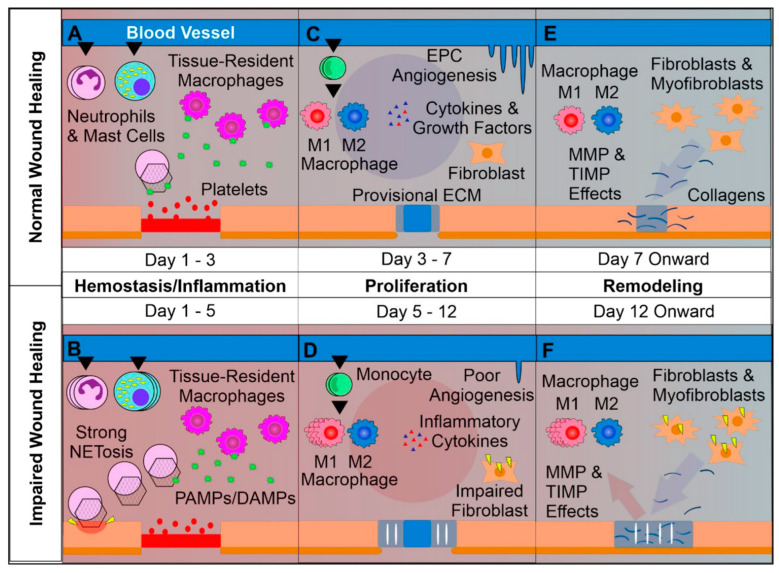
Overview of normal vs. impaired wound healing. (**A**) Platelets form a clot at the site of injury and chemoattractants are released, recruiting key inflammatory cells. Next, inflammation takes charge, with infiltrating neutrophils and mast cells releasing pro-inflammatory cytokines and inducing strong sanitizing effects. This is accompanied by neutrophil extracellular trap (NETosis) induction, which assists in capturing and destroying invading pathogens. Tissue-resident macrophages react to pathogen- and damage-associated molecular patterns (PAMPs and DAMPs), activating. Later a provisional matrix comprised of fibronectin and other provisional extracellular matrix (ECM) components forms from the clot. (**B**) Impaired wounds see an upregulated influx of neutrophils and mast cells, leading to an overactive inflammatory response, causing collateral damage and extending the inflammatory phase to the detriment of subsequent phases. (**C**) Following the resolution of strong inflammation, the proliferative phase begins. Crucially, endothelial progenitor cells are stimulated by growth factors to induce angiogenesis. This angiogenesis allows for wound-resident cells to be supplied with oxygen and nutrients, facilitating their function. Infiltrating monocytes differentiate into M1 and M2 macrophage subsets. M1 macrophages maintain a strong inflammatory profile but are counterbalanced by pro-regenerative M2 macrophages, which release anti-inflammatory cytokines, growth factors and proteases, which replace the provisional ECM with collagens, assisted by properly functioning fibroblasts. This process results in thick granular tissue and full keratinocyte coverage. (**D**) Impaired wounds result in poor angiogenesis and, in the case of T2DM, glycated proteins. This hypoxic environment induces oxidative stress, driving inflammatory M1 macrophage polarization and impairment of fibroblasts, resulting in poor ECM reorganization and a persistent inflammatory environment. (**E**) Remodeling is carried out by macrophages, fibroblasts and myofibroblasts re-organizing the provisional ECM into a coherent scar structure primarily using matrix metalloproteinases (MMPs) and their inhibitors (TIMPs), resulting in tissue with strong tensile strength and functionality. (**F**) Impaired wound-resident cells remain ineffective and pro-inflammatory. Collagen reorganization resolves poorly, resulting in weak, non-functional skin that is apt to re-injure and potentially ulcerate, perpetually inflamed [16].

Wound healing is a natural process via which the body regenerates the dermal and epidermal tissue [2]. It is a common, complex but ordered physiological process that follows the onset of a skin tissue lesion (caused by an external factor like physical trauma, surgery, thermal injury, etc., or an internal factor like disease such as vascular diseases, diabetes, tumor, etc. [5,15]) in order to reconstruct the barrier between the human body and the external environment. It is a dynamic, interactive process that involves soluble mediators, various cell types (e.g., blood cells, parenchymal cells) and extracellular matrix (ECM) and comprises four sequential and overlapping regeneration phases: hemostasis (formation of fibrin plug), inflammation (swelling), proliferation (formation of new tissue and blood vessels) and remodeling of newly formed tissue (Figure 1) [2,15,16,17], which can be affected by specific factors such as wound cause, wound size/depth, disease, age, nutrition, etc. [2].

**Figure 2:**
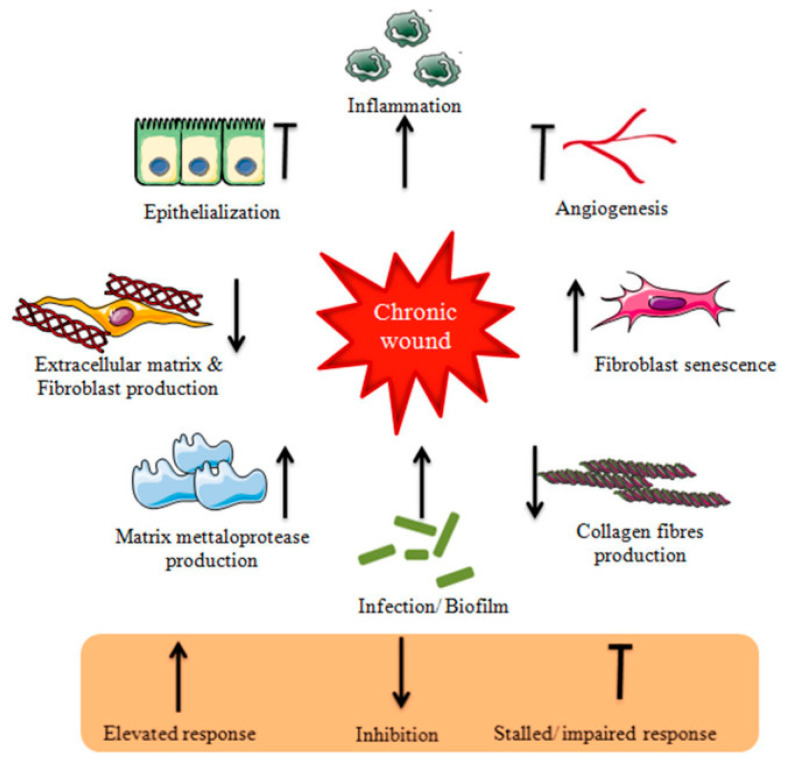
Wound-healing challenges in chronic wound environment [18].

Trauma leads to the immediate activation of a clotting cascade, which results in the formation of a fibrin plug ensuring hemostasis. Platelets trapped in the clot release pro-inflammatory mediators (e.g., growth factors, cytokines) into the local wound environment, thus ensuring the invasion and recruitment of inflammatory (neutrophils, monocytes) and other cells to the wound area. Hemostasis typically lasts 2–3 h and triggers inflammation, which begins when the injured blood vessels leak transudate resulting in swelling. Inflammation controls bleeding and at the same time prevents infection. During this phase, the duration of which can vary from hours to days, pathogens, bacteria and damaged cells are removed from the wound. In the proliferative phase, proliferation and migration of endothelial cells and fibroblasts take place promoting angiogenesis and formation of new ECM. In this way, the wound is rebuilt with new tissue made up of nascent ECM. Furthermore, a new network of blood vessels is created allowing the granulation tissue to receive sufficient oxygen and nutrients. Epithelial cells migrate from wound edges, initiating epithelialization and thus a new epithelial barrier appears. As new tissues are built, the wound contracts. Keratinocyte differentiation aids in restoring the function of the epidermis as a barrier. The remodeling or maturation stage (lasting for months), refers to the phase when the new ECM is constantly reorganized/reconstructed by myofibroblasts. The collagen network is densified by the microfilaments attached to the ECM and the wound is further contracted. Simultaneously, new components are secreted, increasing matrix density and stability. Finally, ECM is further strengthened by fibroblasts and the wound fully closes, usually with the production of disordered tissue [2,4,16,17]. Disturbance of the healing process by microbial invasion or underlying pathological mechanism results in interruption and/or deregulation of one or more of the aforementioned phases and leads to non-healing (chronic) wounds such as diabetic, venous and pressure ulcers [2,4,7,16,17]. The latter are characterized by the accumulation of reactive oxygen species (ROS) and proneness to infection as well as a prominent and prolonged inflammatory phase leading to destruction of ECM and sequential effect on resident fibroblasts. This includes the alteration of fibroblasts’ ability to proliferate and synthesize/remodel ECM since the chronic wound-associated fibroblasts develop a senescent phenotype. Finally, the chronic wound environment impairs angiogenesis and delays epithelialization leading to delayed and/or impaired wound healing [15,18]. Figure 2 shows the abovementioned challenges, which result in retarded wound healing or non-healing.

In the specific case of diabetic ulcers, hyperglycemia and mitochondrial dysfunction lead to permeation of the wound microenvironment by reactive oxygen species (ROS) resulting in persistent and prolonged inflammation and vascular endothelial dysfunction as well as tissue necrosis [9]. More specifically, hypoxia is considered a major reason for wound damage caused by the limited supply of oxygen due to neuropathy and vascular dysfunction, as well as enhanced oxygen consumption by cells at the wound site. Moreover, the disproportion between angiogenic and angiostatic factors can result in angiogenic imbalance and exacerbate wound hypoxia. Hypoxia can also intensify inflammatory responses, thus prolonging injury by enhancing oxygen radicals’ levels. Furthermore, the remodeling phase is hindered due to the considerably reduced proliferation and function of fibroblasts and differentiation into myofibroblasts. Additionally, increased levels of glucose-mediated induction of matrix metalloproteases-9 (MMP-9) overexpression impair the migration of keratinocytes. Finally, enhanced glucose levels can decrease VEGF and HIF-1α activity while increasing non-enzymatic glycation of important proteins resulting in abnormal function of cells and ECM and thus inhibiting angiogenesis [17].

To date, all types of antibiotics have been administered for the treatment of bacterial infections. However, chronic use of antibiotics should be avoided since it leads to drug resistance. Recently, different biomaterials exhibiting superior antibacterial activity have been developed based on metallic ions (e.g., silver and copper ions, zinc oxide, etc.), which, however, could cause serious toxicity. Therefore, the design/development of biomaterials that, apart from hindering mtROS generation and enhancing the supplementation of energy during hyperglycemia, could also sterilize wounds in an efficient and biosafe way, is urgently needed [9]. Furthermore, it has been recently revealed that the depth and duration of wounds can be related to microbial variety/loading. Accordingly, the characterization of the microbiome in order to distinguish between nonthreatening and problematic bioburden and localize bacterial colonies, alongside pathogens, is considered very important [19].

## 3. Wound Dressings

Typical wound treatments comprise surgical (e.g., debridement, skin grafts/flaps), and non-surgical (e.g., topical formulations, skin replacement, wound dressings incorporating or not growth factors, bioactive agents, nanoparticles) methods. Modern approaches include growth factors/cytokine therapy, stem cells (SCs) therapy, vacuum-aided wound closure, and three-dimensional (bio)printed wound dressings. Another approach involves the bioengineering of skin substitutes based on combinations of biomaterials, growth factors and cells [20].

Wound dressings are typically compresses or sterile pads that are applied directly onto the surface of wounds in order to protect them from further injury and promote their healing process. The required characteristics of wound dressings are presented in Table 1. 

Dressings can be categorized into traditional and modern. Traditional dressings such as gauzes (woven and nonwoven), plasters, cotton wool and bandages (natural and synthetic) are economical and are typically used either as primary or secondary dressing products to protect wounds from being infected [2,21]. Due to their fibril-based structure, gauze pads are capable of absorbing wound exudates. However, since they are dry, they tend to stick to granulation tissue and their removal becomes very painful. Accordingly, they have to be frequently changed to avoid infection, and adherence to the wound as a consequence of absorbed exudates and the maceration of neighboring tissue. Furthermore, since they are not hydrophilic, they cannot provide a moist environment for the wound to promote healing. The above have been considered as major drawbacks and, accordingly, traditional dressings have become less desirable for exuding wounds and have been replaced by more advanced modern dressings [6,21,22,23].

The latter have been designed to ease wound healing apart from simply protecting the injured area. Modern dressings can be classified into biological and artificial. Biological dressings comprise donated human, animal or cadaver skin. These “auto-grafting” dressings are most appropriate for the complete healing of chronic deep wounds and/or burns. However, the insufficiency of donor skin is a serious drawback [2]. Artificial dressings are made of natural or synthetic polymers, composites, etc., and are characterized by the presence of a highly absorbing layer and semi-permeability [2,22]. Back in the 1980s, polyurethane (PU) foams and gels containing iodine and hydrocolloids were presented as wound dressings that absorb fluids and provide moisture. In the 1990s, artificial dressings included films, silicone meshes, synthetic foams, hydrocolloids, alginates, hydrogels, vapor-permeating adhesive films, dressings containing silver and collagen, etc. [2]. The above dressings, characterized by increased fluid handling properties, were developed to address the limitations of the traditional dressings, such as the maintenance of ideal humidity and temperature and the alleviation of chronic wound conditions (e.g., presence of increased levels of proinflammatory cytokines) in order to accelerate the healing process (e.g., granulation tissue formation, epithelial cells migration from the wound edges towards the wound center [22]), minimize the risk of maceration and protect the wound from bacterial invasion as well as prevent cross infection [6,21]. Modern dressings may also exhibit anti-inflammatory, antimicrobial, antioxidant and epithelializing properties as a result of their impregnation with pharmacologically active ingredients (e.g., antibiotics, analgesics, anti-inflammatory drugs, natural extracts, etc.) [6].

The selection of the ideal dressing for chronic wounds depends on the physiological conditions of the wound (e.g., location, wound depth and extent, wound adhesion, secreted exudate volume and viscosity, infection, etc. [6,21]). Consequently, it is crucial to select an appropriate dressing to stimulate the healing process [6]. For example, highly exuding wounds should be covered with dressings exhibiting adequate liquid absorption to avoid leakage around or through them. Apart from proper absorption capacity, the dressing should be capable of retaining the absorbed fluid. A balanced moisture wound environment would thus prevent maceration or overhydration and promote healing. At this point, it should be mentioned that the exudate composition varies with wound type. While in acute wounds it promotes wound remodeling and repair, in chronic wounds it slows down wound reconstructing cells’ proliferation because of the increased level of denaturing proteins (e.g., proteases, proinflammatory cytokines). In addition to the balanced moisture wound environment, the dressing should tackle bacterial invasion since highly exuding wounds are considered to be the perfect environment for the growth and spread of bacteria, which hold back the healing process [21].

Modern dressings are considered the top choice for curing various wound types due to their exceptional biocompatibility/biodegradability, ability to maintain a moist environment and temperature, pain relief, and improvement of a hypoxic environment. Those most commonly used in clinical practice are films, foams, hydrocolloids, alginates and hydrogels [6]. Table 2 shows examples of commercially available modern dressings.

### 3.1. Film Dressings

Film dressings are very thin, transparent polyurethane sheets of increased flexibility, which adhere to the periwound skin and achieve maintenance of moisture in the wound environment. They are impermeable to water and microorganisms but permeable to oxygen, water vapors and carbon dioxide. Due to their strong adherence, they can be applied to moving surfaces like joints but they cause pain and damage to the periwound upon removal. On the other hand, excessive accumulation of wound fluid beneath the dressing can result in loosening of the adhesive and leakage of the fluid, leading to maceration and facilitating bacterial invasion. Film dressings are typically applied to superficial and newly healed wounds, including graft sites of split skin and peripheral venous catheter sites [6].

### 3.2. Foam Dressings

Foam dressings most commonly consist of a polyurethane foam covered by a silicone film which acts as a microbial and water barrier. They are highly absorbing, depending on the foam thickness, texture and pore size, and can maintain a moist wound environment. They also provide thermal insulation. Their increased absorption capacity qualifies them for the treatment of exuding wounds. They can be attached to the wound for up to seven days, depending on the volume of the wound exudate. They are usually applied to minimal and moderately exuding wounds, burns, chronic wounds and ulcers. Then again, they are not recommended for the treatment of dry epithelial wounds, necrotic wounds and those needing frequent changes [6].

### 3.3. Hydrocolloid Dressings

Hydrocolloid dressings usually comprise self-adhesive hydrophilic colloid granules (e.g., carboxymethyl cellulose, gelatin, pectin) of various sizes, coated with a water-resistant polyurethane (PU) film protecting the wound from external agents such as bacteria, environmental agents, etc. They are capable of absorbing large amounts of wound exudate while being impermeable to vapors and oxygen, maintaining this way a moist wound environment, stimulating epithelialization and synthesis of collagen, and decreasing the pH of wound fluid resulting in bacteria reduction. In addition, they prevent infection and promote the removal of damaged/infected tissue via autolysis. Moreover, they do not need secondary dressings. They are frequently impregnated with active agents in order to treat lower-extremity and/or pressure ulcers. They are typically applied to low and/or moderate exuding wounds, granular and necrotic wounds, as well as acute wounds such as partial and/or full-thickness burns and (post)surgical wounds [6].

### 3.4. Alginate Dressings

Alginates have been extensively used in wound healing because of their valuable properties, like enhanced absorption capability, biocompatibility, non-toxicity and permeability to gases (e.g., oxygen, etc.) and liquids. They have been manufactured in various wound dressing forms, such as films, porous sheets, nanofibers, hydrogels, etc. [6,67]. In comparison with traditional dressings such as gauze, alginate dressings absorb excess wound exudate while retaining their structural integrity, thus providing a moist wound environment, diminishing bacterial infection and stimulating wound healing [6,67]. Furthermore, they can diminish wound odor and inflammation, and exhibit hemostatic properties [6]. Upon application onto the wound, alginate forms a gel and easily sloughs when removing the dressing or rinsing with sterile saline. A secondary dressing is usually required in order to stabilize the non-adhesive alginate dressing. Alginate is appropriate for the treatment of both acute and chronic exuding wounds like pressure ulcers, diabetic foot ulcers, (infected) surgical wounds and burns [6]. In the absence of adequate liquid necessary to form a gel, alginate could leave fibers at the wound site which could cause inflammation [6]. Alginate dressings are usually combined with various synthetic polymers to increase their mechanical properties. The therapeutic efficiency of the composite dressing is dependent on the ratio of synthetic polymers to alginate, the degree of crosslinking as well as the encapsulation of antimicrobial agents and/or nanoparticles [67].

### 3.5. Hydrogel Dressings

The development of hydrogels as potential wound dressings for pressure ulcers, dry chronic wounds, necrotic wounds, burns, etc., has received a lot of attention because of their three-dimensional (3D) porous structure mimicking extracellular matrix (ECM), their high water absorption and their mechanical properties (e.g., elasticity, softness) providing a cooling/soothing effect and facilitating the dressing application and removal, their oxygen permeability and their ability to encapsulate various active ingredients (e.g., pharmaceutics, growth factors, etc.) [2,5,70]. In particular, injectable hydrogels have triggered research interest due to their ability to fill irregular wounds, thus avoiding gel fragmentation, and their inherent self-healing properties [70].

Both natural (e.g., collagen, chitosan, hyaluronic acid, alginate, gelatin, etc.) and synthetic (e.g., poly(ethylene glycol dimethacrylate), poly(ethylene oxide), poly(hydroxyethyl methacrylate), poly(acrylic acid), etc.) polymers have been used for the formation of hydrogels [5] with a preference towards natural polymers exhibiting biocompatibility, nontoxicity, enhanced cell attachment and strong activity against bacteria [5,70]. Nevertheless, the performance of hydrogels formed using a single natural or synthetic polymer is often limited, so the research interest has focused on the design/development of multifunctional hydrogels with superior properties to be applied as wound dressings [2,5], including hydrogels efficiently encapsulating conductive agents, which further promote wound healing via regulation of cell activities like adhesion, proliferation and migration [70], as well as pharmaceutics, bioactive agents and/or nanoparticles [5]. It should be pointed out that all hydrogel properties (e.g., physicochemical, rheological/mechanical, biological) can be readily affected by their chemical composition (i.e., selection of polymer backbones, functional groups, crosslinking mechanism and secondary crosslinking interactions, as well as integration of nanocomposites) resulting in the formation of hydrogels exhibiting critical characteristics like injectability, stimuli responsiveness, self-healing, etc. [70]. 

**Figure 3:**
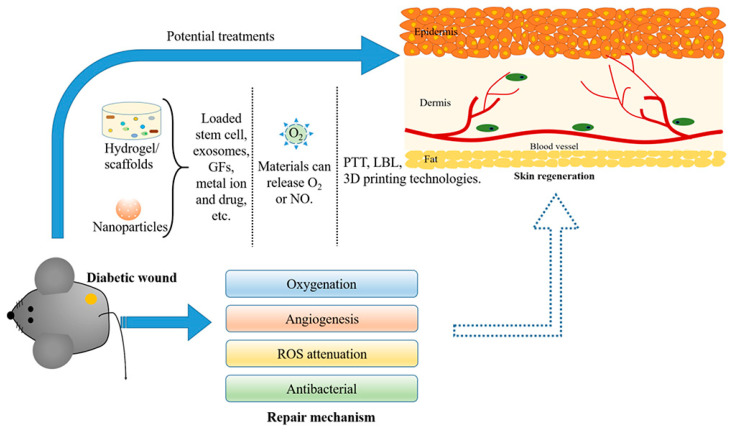
Potential therapies for diabetic wound repair. Strategies for manipulating the regeneration of diabetic wounds include the use of hydrogels (loaded with small molecules and stem cells, etc.), photothermal therapy and materials that release oxygen. All of these elements have been demonstrated to have an effect on in vitro and in vivo models of wound healing. These repair mechanisms include vascularization, less ROS production, oxygen release and antimicrobial resistance. Therefore, combining these strategies will undoubtedly change the result of diabetic wound healing [17].

In contrast, with the aforementioned modern wound dressings (e.g., films, foams, hydrocolloids), hydrogels exhibit easily tuned degradation properties, which make them suitable candidates for applications requiring targeted delivery of bioactive agents (e.g., growth factors, proteins/peptides, genes, stem cells/exosomes, etc.) and/or non-bioactive agents (e.g., oxygen, metal ions, nitric oxide, etc.) to the wound site (Figure 3) [17,22]. In this respect, the design/development of novel injectable multifunctional hydrogels encapsulating various types of bioactive agents and exhibiting self-healing, antibacterial activity and multi-stimuli responsiveness, via advanced technologies like electrospinning and 3D (bio)printing has recently become a hot research topic [5,17]. It should be noted that antimicrobial hydrogels (AMHs) are divided into hydrogels encapsulating antimicrobial agents and those with inherent antimicrobial properties [71].

### 3.6. Cell-Based Dressings

Cell-based dressings have been assessed and commercialized (Table 3) with an aim to replace the current standard of care (SOC) for complex/chronic wounds, which involves wound moisture control via cautious selection of appropriate wound dressings, providing the conditions for accurate protein/cell interactions that would promote wound healing. They typically use a cell-seeded hydrogel scaffold and their wound healing efficiencies (even though improved in comparison with SOC) vary between 31 and 50% of wound closure. Apart from their limited efficacy, their clinical translation is limited by higher upfront costs. The above could be overcome through the implementation of innovative manufacturing technologies like 3D bioprinting [4].

## 4. Three-Dimensional Printing

Three-dimensional (3D) printing is a well-known method employed to fabricate accurately designed 3D architectures with high resolution based on computer-aided design (CAD) and with the use of biocompatible inks (i.e., biomaterials that can be 3D printed). To be sure, 3D printing enables the fabrication of scaffolds of specific shapes, exhibiting controlled porous structure, permeability, mechanical properties, etc., for tissue engineering (TE) applications (e.g., tissue regeneration, engineering of artificial organs, etc.) [20,70,72,73,74]. The 3D-printed porous constructs promote oxygen exchange and ease the removal of metabolites, improving this way of cell proliferation [70]. The introduction of 3D printing to wound dressings has revealed promising results as a consequence of the method’s capability for controlled design and fabrication of dressings exhibiting tuned microstructure [8]. Furthermore, it allows the temporally and spatially controlled release of various bioactive agents (e.g., drugs, growth factors, antimicrobial agents, nanoparticles, etc.) [5,74,75].

### 3D-Printed Hydrogels

Hydrogels are a popular class of biomaterial inks owing to their biomimetic properties and their benign processing conditions entitling them as suitable candidates for TE applications. They are usually printed in the form of their precursor materials and their final structure is obtained via crosslinking during or post 3D printing [73]. Shape fidelity and collapsing are typical challenges in 3D hydrogel printing related to the viscoelastic properties of the ink and its solid content, respectively. Ideally, the ink should be able to flow through the nozzle throughout the printing process and preserve its shape post printing [76]. Hydrogel precursors need to have a suitable viscosity to preserve their structural integrity until crosslinking. This can be facilitated by the increase in the polymer concentration, the addition of composites and the use of (near) gel-phase inks such as gelatin solutions which can be printed at a temperature close to their sol–gel transition (near gel-phase inks) and partially crosslinked hydrogels like alginate solutions containing low concentrations of calcium chloride (gel-phase inks) [73], as well as via the use of rheology modifiers such as cellulose nanofibrils, which could improve ink printability and achieve shape fidelity post printing [76].

Recent advances in 3D printing technologies (e.g., extrusion-based 3D printing) permit hydrogel customization according to wound size and depth [13,14] and enable the formation of multi-component hydrogels exhibiting various microstructures and networks of interconnected pores which facilitate the transport of oxygen, nutrients, metabolic wastes, etc. [13,14], as well as the temporal and spatial control of bioactive’s release thus promoting bacteria reduction, favoring tissue proliferation and decreasing healing time [14].

Various types of polymers, both natural (e.g., sodium alginate (SA), chitosan, gelatin, carboxymethyl cellulose (CMC-Na), etc. [74,77]) and synthetic (e.g., 4-arm PEG [73], 2-hydroxyethyl methacrylate (HEMA), polyethylene glycol dimethacrylate (PEGDA), poly(acrylic acid) (PAA) [78], etc.), as well as combinations thereof, have been used for the formation of hydrogel inks. From a chemical point of view, the selected materials should be easily modified with various chemical groups in order to be crosslinked via different mechanisms (e.g., free radical, ionic, etc.) and functionalized with appropriate molecules (e.g., functional polymers, adhesion peptides, peptides cleavable by proteases). They should also undergo hydrolysis and/or enzymatic degradation, potentially exhibiting inherent antibacterial properties, stimuli responsiveness, etc. Finally, they could permit the formation of a reversible 3D network via dynamic chemical bonding to enable self-healing [5].

**Figure 4:**
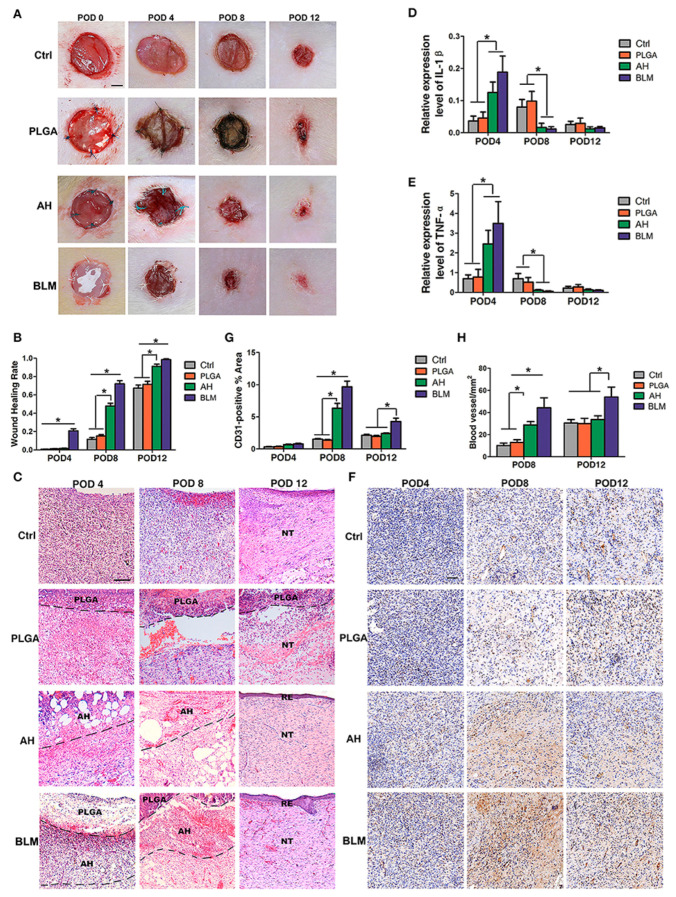
In vivo wound healing and staining. (**A**) In vivo wound healing of blank control, PLGA, alginate hydrogel (AH), and BLM scaffold on PODs 0, 4, 8 and 12; NT, new tissue; RE, re-epithelization (Scale bar 250 μm). (**B**) Analysis of wound healing rates in different groups. (**C**) H&E staining of control, PLGA, AH, and BLM scaffold on PODs 4, 8 and 12 (Scale bar 50 μm). (**D**) Q-PCR analysis of IL-1b in different groups on PODs 4, 8 and 12. (**E**) Q-PCR analysis of TNF-a in different groups on PODs 4, 8 and 12. (**F**) Immunohistochemical staining of different groups on PODs 4, 8 and 12 (Scale bar 5 μm). The red arrow indicates the vessels. (**G**) Quantification of blood vessel density in CD31-stained tissue sections. (**H**) Quantitative analysis of CD31-positive area (* *p* < 0.05) [81].

Sodium alginate (SA), a cost-effective marine-derived polysaccharide, characterized by excellent biocompatibility, enhanced aqueous solubility and minimal toxicity, has been widely utilized in 3D printing of wound dressings since it can rapidly crosslink with divalent cations (e.g., Ca^2+^, Mn^2+^, Ba^2+^, Cu^2+^) and absorbs excess wound fluid while it maintains a physiological moist wound environment [13,77,79,80]. On the other hand, shape infidelity, excessive swelling, rapid degradation rate, low mechanical properties, etc., could hinder its use in the 3D printing of wound dressings [77,79,80]. Nevertheless, modification and/or enhancement of SA by combination with different organic/inorganic materials (e.g., various polymers such as gellan gum [13], collagen, gelatin, cellulose, etc., and/or nanoparticles) can improve its performance [77,79]. In this respect, Wang and coworkers (2019) designed and fabricated a bilayer membrane (BLM) comprising a layer of alginate hydrogel printed on a poly (lactic-co-glycolic acid) (PLGA) fibrous membrane mimicking the dermis and epidermis, respectively. The PLGA membrane was found to prevent bacterial invasion and maintain the hydrogel moisture content, while the hydrogel layer stimulated cell adhesion and proliferation. In vivo application of the bilayer dressing resulted in the deposition of collagen I/III and enhanced neovascularization leading to skin regeneration (Figure 4) [81].

**Figure 5:**
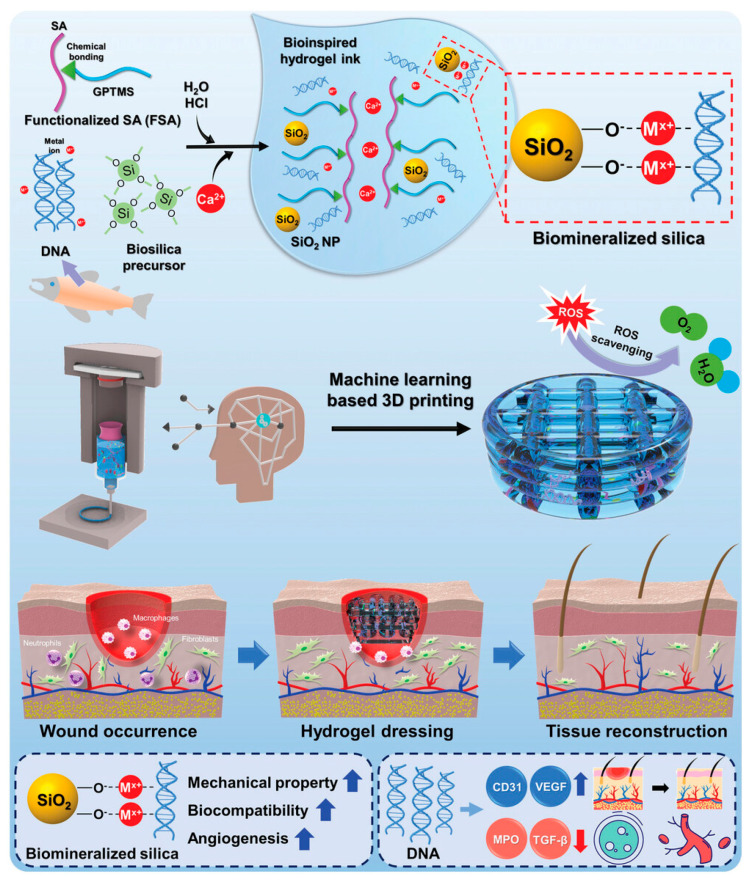
Schematic diagram of the fabrication of bioinspired 3D-printed hydrogels using DNA-induced biomineralization [79].

In a very recent work, Kim and coworkers (2023) developed a bioactive alginate hydrogel ink incorporating salmon sperm-derived DNA and DNA-induced biomineralized silica and exhibiting biocompatibility, printability, mechanical stability as well as a reactive oxygen species (ROS) scavenging effect for machine learning-assisted 3D printing of hydrogel wound dressings (Figure 5). The fabricated hydrogel dressing was shown to have suitable porosity and mechanical properties and to efficiently absorb blood and wound exudate. Moreover, both DNA and biosilica enhanced the bioactivity of the hydrogel dressing regarding ROS scavenging, anti-inflammatory activity and angiogenesis, thus accelerating wound healing in acute and diabetic wounds [79]. In another study, Shen and coworkers (2021) developed an antibacterial hydrogel wound dressing of alginate embedded with the locally isolated bacteriophage (HZJ), which targets H5α *Escherichia coli*. The printed hydrogel dressing was revealed to gradually release lytic phages and to efficiently suppress bacterial growth for up to 24 h, thus responding to the rise of drug-resistant bacteria [82]. To treat wounds exhibiting bacterial infection, Chen and coworkers (2023) printed via co-axial 3D printing a hydrogel scaffold comprising dopamine-modified alginate and gelatin, which was crosslinked with Ca^2+^/Cu^2+^. Apart from being released from the hydrogel in a sustained manner, Cu^2+^ endowed the construct with a photothermal effect, which further improved its antibacterial activity on both *S. aureus* and *E. coli.* Furthermore, the released Cu^2+^ in combination with the hollow channels was shown to promote angiogenesis and wound healing [83].

Gelatin, a low-cost, collagen-derived biocompatible/biodegradable material containing an RGD (i.e., arginine−glycine−aspartic acid) adhesive peptide sequence supporting cell adhesion, and matrix metalloproteinase (MMP) cleavage sites enabling its degradation in the presence of MMPs, has been also used for the development of inks but combined with other polymers due to its insufficient mechanical properties [84,85]. Gelatin methacrylate (GelMA) is also considered a promising option for the development of 3D-printed wound dressings because of its tunable properties controlled by the degree of methacrylation, as well as its ability to be photocrosslinked [72]. On the other hand, its poor mechanical properties could restrict its clinical application [86]. Chondroitin sulfate (CHS) is a glycosaminoglycan that has an essential role in ECM and cartilage, as well as in wound healing, growth factor signaling and inflammation. Nonetheless, it is difficult to form CHS-based materials for tissue regeneration through physical/chemical crosslinking. Remarkably, CHS methacryloyl (CHSMA), formed via the introduction of photocrosslinkable methacryloyl groups into the CHS chain is broadly used in TE applications [80].

Low-cost 3D-printed chitosan hydrogels have also attracted a lot of interest owing to their significant biocompatibility, biodegradability, absence of toxicity and antibacterial activity. On the other hand, its potential relation with toxic organic solvents and its poor mechanical properties (i.e., chitosan is very soft and thus deforms or collapses post printing) could limit its application in TE [87]. Therefore, it should be reinforced with other materials such as (bio)polymers in order to be successfully printed and preserve the scaffold shape [88]. In this framework, Zarandona and coworkers (2021) reinforced chitosan hydrogels with the abundant, biodegradable and non-toxic anionic heteropolysaccharide pectin which forms polyelectrolyte complexes with chitosan. The printed hydrogels were reported to exhibit a high degree of swelling and appropriate strength for shape maintenance after compression indicating their potential to be used as wound dressings [88]. In another study, Zhou and coworkers (2020) printed a chitosan ink comprising a chitosan solution (i.e., chitosan dissolved in an alkali aqueous solution) through extrusion into warm water where it crosslinked in situ by temperature increase via self-assembly of the polymer chains. This direct ink writing (DIW) method allowed the 3D printing of chitosan hydrogels of high quality and strength (e.g., 2.31 MPa for compression) suitable for wound healing applications [87].

Cellulose-derived materials have been found to be promising for the fabrication of scaffolds mimicking ECM due to their biocompatibility, non-cytotoxicity, porous microstructure, favorite mechanical properties and tunable architecture, despite their challenging degradability in vivo as a result of the absence of relevant enzymes [74]. Bacterial cellulose has been widely studied and used in various commercial applications. Yet, because of the nature of its cultivation, post-printing shaping remains challenging. The more shapeable wood-derived nanocellulose has been shown to be biocompatible and to support important cellular processes during the culture of various cell lines. Nanocellulose could be used for the development of inks because of its mechanical strength and mimicry of ECM structure provided that the stability issues of the printed scaffold can be surpassed (e.g., via double crosslinking [74] and/or the use of an auxiliary polymer [89]) [74]. Carboxymethyl cellulose (CMC), a cellulose derivative has been used in wound healing applications as hydrogel dressing because of its biocompatibility, good mechanical properties and increased stability. CMC has been also combined with ε-Polylysine (ε-PL), a sort of cationic polypeptide exhibiting antibacterial properties but poor mechanical properties, for the 3D printing of hydrogel dressings having an ordered porous structure and antibacterial/antioxidant properties, suitable for large irregular wounds [90]. Cellulose nanofibrils (CNF) could be used as rheology modifiers for improved printability and maintenance of shape fidelity post printing. Finally, cellulose nanocrystals (CNC) could be used as ink components and/or reinforcement for alginate hydrogels [76].

Temperature-stimulated polymers such as poly(N-isopropylacrylamide) (PNIPAAm), capable of gelling at body temperature due to their low critical solution temperature (LCST) have been widely studied as potential materials for wound dressings. Furthermore, PNIPAAm-based hybrid networks have exhibited potential as injectable materials for wound closure [91]. Interpenetrating polymer networks, also known as superporous hydrogels, formed via the reaction of ethylene monomers such as N-isopropylacrylamide, acrylic acid, acrylamide, vinylpyrrolidone, etc., with natural polysaccharides and/or polymers containing hydroxyl or carboxyl groups are mostly attractive for the management of chronic wounds due to their increased porosity and cell adhesion. Nonetheless, typical superporous hydrogels lack antibacterial properties and enhanced swelling following the uptake of liquids [92]. Wu and Hong (2019) overcame this problem by combining 3D printing with the silver-ethylene interaction to print nanocomposite supeporous hydrogels of polyacrylamide (PAM) and hydroxypropyl methylcellulose (HPMC) crosslinked with Ag NPs as potential wound dressings. The hydrogels were revealed to stimulate infected wound healing and restrain the formation of scar tissue [93]. In another study, Niziol and coworkers (2021) designed an ink comprising PNIPAAm precursors, methylcellulose (MC) and sodium alginate (ALG) and containing 2-phenoxyethanol (Octenisept^®^, OCT) and octenidine dihydrochloride, which enabled the accurate printing of a non-cytotoxic temperature-responsive hydrogel exhibiting shape fidelity and antimicrobial activity against Gram-positive and Gram-negative bacteria, as well as fungi [91].

The autologous platelet-rich plasma (PRP) gel, rich in growth factors such as vascular endothelial growth factor (VEGF), platelet-derived growth factor (PDGF), epidermal growth factor (EGF), etc., is considered to be a promising approach for the treatment of diabetic foot ulcers due to absence of immunological reaction, acceleration of cell proliferation and wound tissues’ migration. Still, PRP gel undergoes rapid growth factor (GF) release, thus requiring repeated administration resulting in retarded wound healing, costly treatment and increased patient suffering [93]. To resolve this problem, Huang and coworkers (2023) developed multi-layered, fibrous, core–shell, bioactive hydrogels loaded with PRP via coaxial microfluidic 3D bioprinting [93]. Integration of microfluidics with 3D printing technology allows the precise control of the structure and composition of engineered constructs (e.g., biomimetic, vascular-like channel structures) promoting vascular network formation and tissue regeneration [94]. The formed hydrogels were revealed to be biocompatible, possess remarkable water absorption/retention properties and antibacterial activity, as well as enable the sustained release of the growth factors and promote cell adhesion, orientation, growth and proliferation. Additionally, the hydrogels were shown to support granulation tissue growth, angiogenesis and formation of a high-density, ordered network of collagen fibers, thus proving their ability to be used as dressings for the treatment of diabetic wounds [93].

Recently, ECM-based bioinks have received a lot of attention for the 3D printing of biomimicking tissue constructs. Due to the lack of organ availability, ECM of human placental has been considered as an alternative source for the 3D printing of composite constructs for treating deep wounds [95]. In a recent study, placenta-derived ECM was combined with sodium alginate and gelatin to form a natural skin-mimicking printable ink. Upon application to a full-thickness wound in a mouse model, the printed construct was shown to enhance the formation of granulation tissue, re-epithelialization and angiogenesis in comparison with printed constructs that did not contain ECM [95].

Various nanoparticles and composites can be incorporated in hydrogels as smart delivery systems because of their exceptional physical properties (e.g., high surface area, response to external stimuli like temperature, light, electrical signal, etc., and controlled release of bioactive agents such as growth factors). Nevertheless, their performance can be hindered due to their uptake by cells via endocytosis and subsequent cell damage by increased ROS concentration, etc. The latter could be counteracted by the substitution of nanoparticles with nanostructured microparticles, as in the work of Siebert and coworkers (2021) who substituted the cytotoxic ZnO NPs with the tetrapod-shaped ZnO particles, which preserve their antibacterial activity without being uptaken by cells through endocytosis and are thus suitable for encapsulation in hydrogels for TE applications [96]. In the same way, Monavari and coworkers 3D printed a hydrogel dressing consisting of alginate dialdehyde and gelatin (ADA-GEL) and containing astaxanthin (ASX) and borate bioactive glass (BBG) microparticles. The latter resulted in the formation of a mechanically robust construct achieving increased viability of fibroblasts and migration of keratinocytes [84].

**Figure 6:**
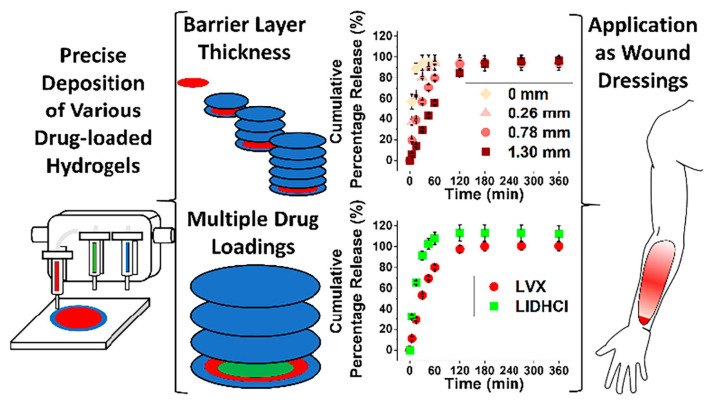
Fabrication of hydrogel wound dressings with customizable architecture and drug loading via implementation of 3D hydrogel printing with permission from [97].

Research effort has also been placed on the implementation of 3D printing for the fabrication of scalable hydrogel wound dressings with customizable drug dosages in the framework of personalized medicine (Figure 6) [97].

In this respect, Alizadehgiashi and coworkers (2021) printed bionanocomposite hydrogels of chitosan methacrylamide and cellulose nanocrystals (CNCs) exhibiting multifunctionality and tunable composition for controlled release of growth factors (e.g., VEGF) and antimicrobial agents (e.g., silver NPs). The dressings were printed in a mesh-type format, where different agents were encapsulated in distinct compartments of the dressings and their release was controlled via variation of compartments’ volume. The developed versatile wound dressings could induce a variation of responses in vivo and could be further adapted for patient-specific treatment of various types of wounds [75].

**Figure 7:**
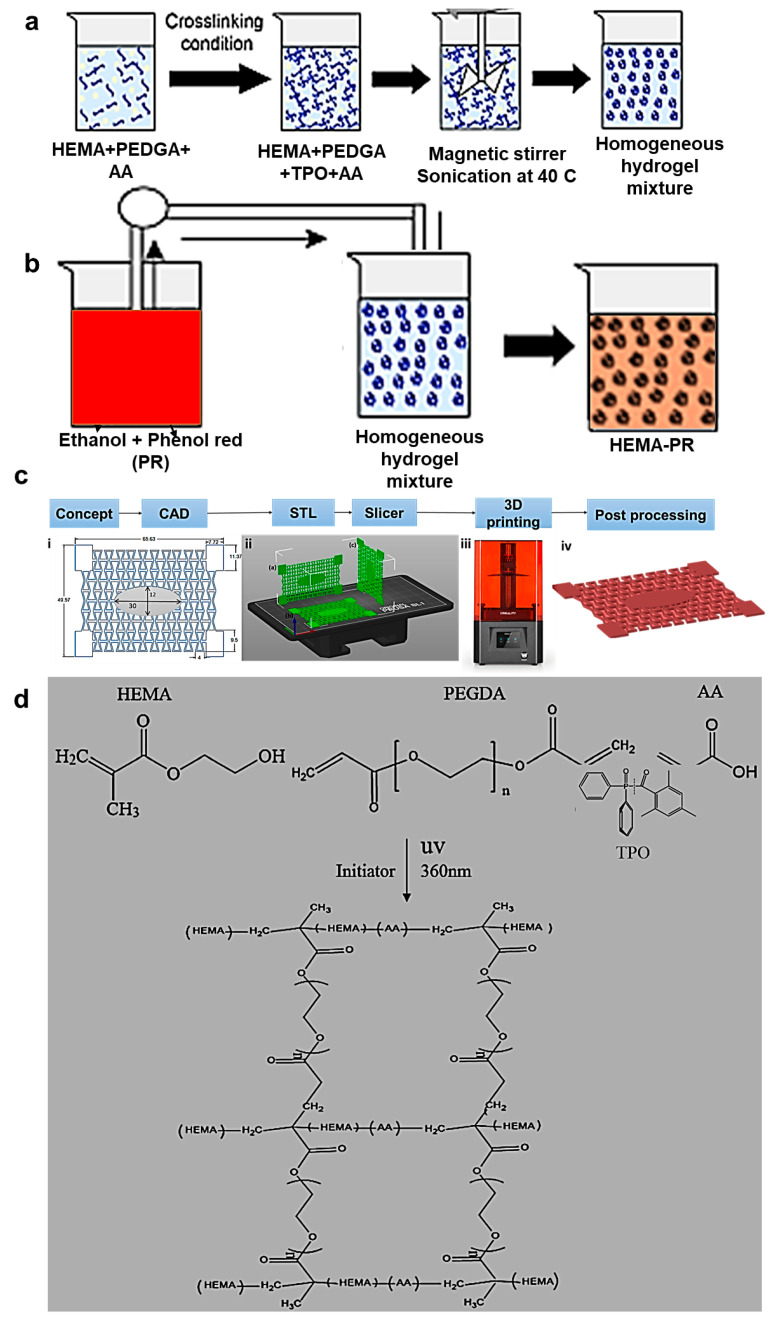
(**a**) Hydrogel resin preparation; (**b**) dissolving of phenol red (PR) in ethanol; (**c**) additive manufacturing process from initial concept to the final printed hydrogel wound dressing; (**d**) chemical synthesis of the HEMA/PEGDA/TPO/AA [78].

Finally, various research studies have described the embedding of pH sensors into 3D-printed hydrogel dressings. External biosensors such as electronics or biomarkers incorporated in the dressings should be biocompatible, non-toxic and should have similar flexibility and stretchability with the hydrogel network applied to the body. In addition, they should be responding to hyper-inflammation and/or possible wound infections and should degrade at a rate proportional to that of the hydrogel dressing. The major limitations encountered in the integration of biosensors in the hydrogels encompass the additional costs and the complexity of the process. To overcome the aforementioned challenges, the use of non-cytotoxic and cost-efficient dyes such as phenol red has been examined [78]. In this respect, Tsegay and coworkers (2023) developed an auxetic hydrogel wound dressing of HEMA/PEGDA/TPO/AA-PR doped with phenol red as pH indicator (Figure 7) to be applied to moving joints of the body (e.g., wrist joint) and a PAA-based adhesive for the printed hydrogel [78].

Table 4 presents recent results on the preclinical assessment (i.e., in vivo and in vitro testing, as well as physicochemical characterization) of 3D-printed hydrogel wound dressings.

## 5. Three-Dimensional Bioprinting

**Figure 8:**
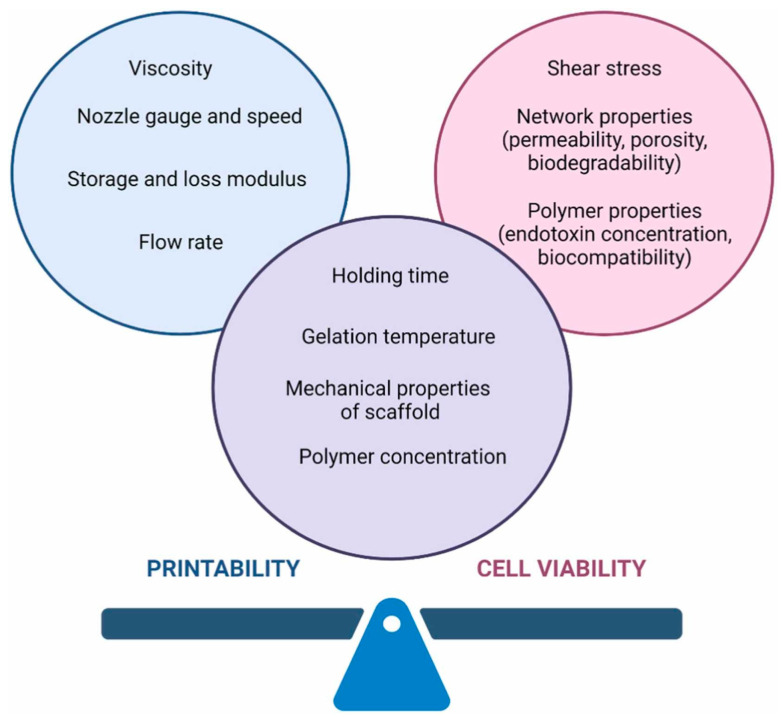
The main challenge in the bioprinting process is to obtain a material with the highest printability, providing good cell viability. Both parameters may be influenced by many factors [135].

Three-dimensional bioprinting, a subfield of 3D printing, is an emergent adaptive bio-manufacturing technology for the accurate fabrication of complex topological constructs based on computer-aided design (CAD), which has been broadly applied to TE, modeling of organoids, etc. It involves the development of bioinks (i.e., biomaterial formulations that contain cells and bioactive agents such as growth factors and can be readily processed by an automated biofabrication technology [133,134,135]) to engineer biologically appropriate constructs mimicking and restoring natural ECM [136]. Bioinks have a fundamental role in the successful fabrication of a skin tissue construct. Their physicochemical/mechanical properties and their composition need to be carefully selected in order to achieve high printability and high cell viability (Figure 8) and to assist cellular functions [135,137]. Bioinks need to be biocompatible to ease cell growth, exhibit controllable rheological properties (e.g., shear thinning) in order to flow effortlessly through the nozzle and possess enhanced shape fidelity after printing (i.e., redeem sufficient storage modulus after printing), biofunctionality to meet biomimicry requirements (i.e., they should mimic ECM regarding biochemical/biomechanical properties [136]) and mechanical stability [23,70,134,138,139], which can be difficult to accomplish with a single-component bioink [134]. Other important parameters that should be taken into consideration for the design and development of bioinks are their compatibility with various printing techniques, printing resolution, bioprintability, biodegradability, maintenance of cell viability and process scalability [137]. There exist various types of bioinks, like cell-laden hydrogels, cell aggregates, etc. Those based on polymers usually comprise natural polymers such as alginate, gelatin, collagen, hyaluronic acid, silk fibroin, fibrin, etc., and/or synthetic polymers like polylactic acid, polyethylene glycol, etc. [133,138]. They are crucial for the printing of constructs with high resolution, good mechanical properties and increased structure stability, as well as the regulation of cell adhesion, proliferation, migration and differentiation [138].

The 3D bioprinting process applied for the manufacture of a wound dressing is similar to the traditional 3D printing process. More specifically, the methods of computed tomography (CT) and/or magnetic resonance imaging (MRI) are utilized to scan the wound site and the images are converted to a CAD model [8]. This part of the bioprinting process is followed by the selection of the appropriate bioink, which is the principal component for the fabrication of functional tissues [8,140], the bioprinting method (e.g., inkjet, micro-extrusion, laser assisted, etc.), the stabilization of the dressing shape via crosslinking (e.g., photopolymerization, etc.) and the direct application to the wound [8]. Traditionally, 3D bioprinting is performed based on plane layering (i.e., on flat substrates). Nonetheless, in real clinical practice, wounds are commonly irregular and uneven [134]. Curvilinear bioprinting enables bioink printing on curved surfaces, resulting in the manufacturing of more irregular/complicated constructs in comparison with typical planar bioprinting. These constructs are characterized by enhanced structural integrity due to the continuity of filaments and the presence of inter-filament bonds in shell-like curvilinear structures. It has been reported that skin, engineered via curvilinear bioprinting with an appropriate bioink, exhibits increased structural integrity for the treatment of irregular cutaneous wounds [141]. In situ bioprinting allows for the direct deposition of biomaterials and cells at the wound site following wound debridement and scanning of its morphological features, thus permitting individualized wound treatment. In a surgical setting, it could enable real-time bioprinting directly at the patient’s wound with increased anatomical accuracy, thus enabling the creation of customized constructs conforming to the unique wound topology. However, it should be noted that the bioink selection criteria for this approach would be further strict [134]. According to the above, the development of an optimal bioink for the biofabrication of skin is a rather challenging task.

Table 5 presents the technical challenges related to 3D bioprinting.

Today, a novel era of bioprinting has emerged, namely 4D bioprinting, which involves smart polymers that change/reform with time in response to external stimuli and thus add a fourth dimension (e.g., time) to bioprinting, aiming to address some of the existing limitations of 3D bioprinting. Moreover, 4D bioprinting is still in its infancy and more research work is needed to elucidate it [142].

### 5.1. 3D-Bioprinted Hydrogels

Hydrogels are the preferred category of bioinks for the in vitro development of various tissues such as skin, cartilage, bone, cardiac, etc., as well as vascularized tissues, due to their biocompatibility/biodegradability, superb bio-adaptability, mimicry of ECM, enhanced water content, and effortlessly tunable 3D structure [70,143]. To date, various hydrogels based on natural and/or synthetic polymers (e.g., hyaluronic acid, chitosan, alginic acid, agarose, PEG, etc. [144]) have been used as bioinks with the majority of them being based on natural polymers. The latter are characterized by the ability to mimic ECM and thus aid cell attachment and proliferation, as well as cell migration and differentiation, counteracting, in this way, their poor mechanical properties [23]. Bioink integrity strongly depends on various parameters such as physicochemical (e.g., viscosity, shear thinning) and printing (e.g., diameter and temperature of nozzle, feed rate, duration of printing) parameters, as well as on cell type, density, etc. Cell selection and sourcing are also crucial to prevent immune rejection following implantation. For example, skin primary cells (e.g., fibroblasts, keratinocytes and melanocytes) can be isolated from the skin of donors and then be co-cultured for skin bioprinting [23].

An important advantage of hydrogel bioprinting, in comparison with traditional fabrication methods, is that it enables accurate control over spatial patterning and construct architecture of individual bioinks containing cellular and biochemical components, thus allowing the replication of biological tissues’ native spatial organization [15]. The method of 3D bioprinting has been applied for wound healing and skin tissue repair since 2012. Initially, collagen bioinks were used followed by various natural-based, individual or composite bioinks, aiming to accelerate wound healing and restore skin integrity due to exceptional biocompatibility, the resemblance of skin ECM and great printability [23]. Conductive polymers (e.g., polyaniline, etc.) have also emerged as an innovative strategy for the development of bioinks that could promote wound healing via electrical stimulation of the wound bed. The latter has been found to facilitate cell migration, proliferation, and differentiation, increase angiogenesis and modulate inflammatory responses, thus speeding up wound healing. Additionally, conductive hydrogels (CHs) could act as platforms for stimuli-responsive delivery of bioactive agents like growth factors, therapeutic molecules, cytokines, etc. Finally, CHs could regulate immune responses during the wound-healing process [145].

The healing of acute wounds is frequently marked by scarring; that is, primarily caused by excessive deposition of pro-fibrotic collagen throughout the proliferative stage. The resultant scar tissue is slightly functional in comparison with healthy skin tissue and exhibits poorer mechanical properties. On the other hand, chronic wounds are characterized by a lack of dermal regeneration as a result of persistent inflammation, diabetic conditions, oxidative stress, accumulation of necrotic tissue and other factors inhibiting the typical process of wound healing. Therefore, the microenvironments of acute and chronic wounds are very different and require the unique designs of bioprinted hydrogels [15]. More specifically, in the case of acute wounds, hydrogels need to cautiously balance skin growth (proliferation of local cells, deposition of ECM) promotion with inhibition of pro-fibrotic processes (local inflammation, contraction of the wound, etc.). In this respect, hydrogels for the treatment of acute wounds often comprise skin-derived biopolymers, different angiogenic factors and fibrosis inhibitors. In contrast, the design of 3D-bioprinted hydrogels for the treatment of chronic wounds focuses on multifunctional constructs exhibiting regenerative, antioxidant and antibacterial properties [15].

#### 5.1.1. Bioinks

Naturally derived biomaterials like collagen, gelatin, chitosan, etc., are generally used in TE applications since they are biocompatible/biodegradable and stimulate cell interactions. On the other hand, uncontrolled degradation, low mechanical strength and possible immunogenicity limit their broad application. To address these challenges, chemical modifications of these polymers have been performed in various cases [100]. Typically, an ideal bioink should contain cell anchoring ligands and exhibit appropriate stability and elasticity for the in vitro maturation period [136]. Among the abundant biomaterials, sodium alginate (SA) and gelatin (Gel) have received broad attention for the development of bioinks [134].

Gelatin (Gel), a natural biopolymer produced by collagen hydrolysis, has received a lot of interest as a bioink component due to its hygroscopic nature, cytocompatibility, plentiful cell recognition sites and thermosensitivity, allowing viscosity adjustment to favor printability. On the other hand, its low printing resolution, poor mechanical properties and fast biodegradation, in the presence or not of enzymes, drastically limits its broad application [138,141]. The methacrylation of gelatin gives an easily photocrosslinkable, non-immunogenic derivative (methacrylated gelatin, GelMA) with tunable biodegradation that has been widely used for the fabrication of constructs. Nonetheless, GelMA chain-growth polymerization mediated by free radicals might result in ROS accumulation and oxygen inhibition, leading to partial crosslinking and reduced cell activity. Moreover, GelMA-based structures are characterized by insufficient mechanical properties [138]. On the other hand, gelatin, chemically modified with glycidyl methacrylate (Gel-GMA), has numerous properties suitable for 3D bioprinting, such as easy photocrosslinking, increased cell viability, enhancement of cell binding and cell-mediated hydrogel degradation, whereas it is characterized by poor tensile strength [146]. Accordingly, there is a necessity for the development of new bioinks to surpass the aforementioned problems.

Photocrosslinkable gelatin-methacryloyl (GelMa), produced via the reaction of gelatin with methacrylic anhydride and the introduction of substituent methacryloyl groups onto the reactive amine has also received a lot of attention for TE applications, owing to its biocompatibility and tunable mechanical properties. Lee and coworkers (2022) developed a GelMA-based bioink containing various agents for skin TE. The bioink was shown to exhibit exceptional biocompatibility and bioactivity because of increased cell adhesion and proliferation, and enhanced expression of biomarkers related to ECM remodeling (e.g., MMP9, Ki67 and decorin) [100].

Alginate, an algae-extracted linear polysaccharide, is characterized by hydrophilicity, biocompatibility/biodegradability, porosity, nontoxicity and facile functionalization, as well as lack of adequate biological cues for adhesion and proliferation of cells [138,143,147]. Its combination with gelatin leads to the formation of semi-interpenetrating networks mimicking ECM structure, which provide sufficient cell adhesion sites facilitating hydrogel–cell interactions. More notably, the secondary covalent crosslinks between the backbone of the alginic acid and the divalent cations give them adjustable structure and stiffness, thus broadening their applications in TE [147]. Hence, this combination is considered ideal for the preparation of intertwined networks, to overcome the drawbacks of the protein-based hydrogels [138]. It should be noted that the combinations of gelatin/sodium alginate hydrogels are more commonly used in extrusion bioprinting [133].

Silk fibroin (SF), the main protein of silk, a natural biomimetic fibrous polymer is characterized by biocompatibility (high cell proliferation and adherence, low inflammation) and biodegradability as well as increased tensile strength. It can be chemically modified by glycidyl methacrylate (Silk-GMA) and bioprinted into different structures (e.g., hydrogel, membrane, porous sponges, etc.) via photopolymerization and aqueous processing. SF bioprinting has been found to result in stable porous structures favoring cell adhesion and growth [146].

**Figure 9:**
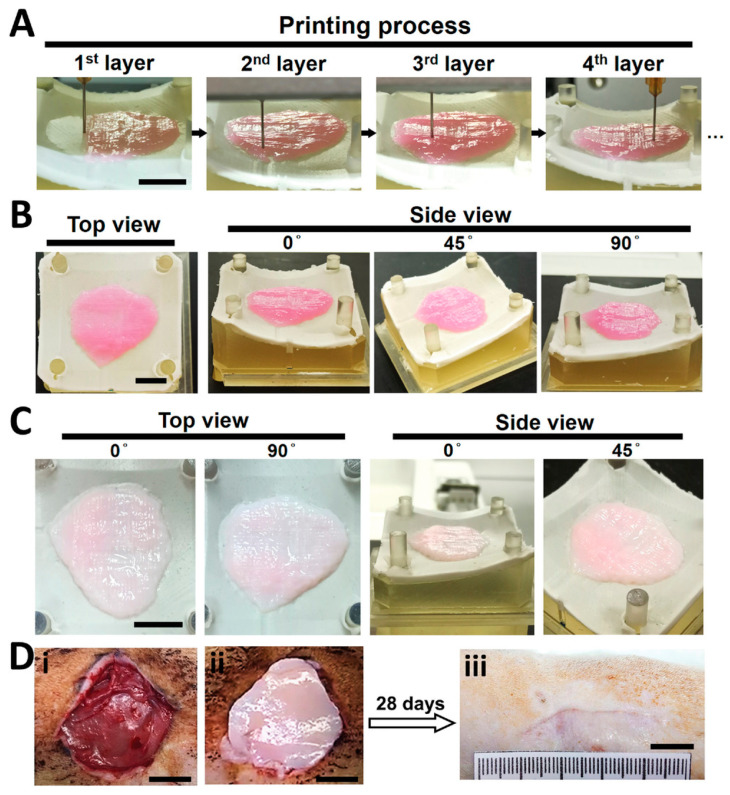
3D curvilinear-bioprinting of the tri-cell-laden PU-gelatin bioink for in vivo wound healing. Photo images show the (**A**) bioprinting process (1st layer to 4th layer) through a 340 μm nozzle, (**B**) bioprinted constructs, and (**C**) constructs after 0.3 N CaCl_2_ treatment for 15 min. (**D**) For healing of the irregular rat skin wounds, a large wound (maximum width of ≈28 mm) was created (**i**) where the construct was implanted (**ii**), and the wound was healed after 28 days (**iii**). Scale bars represent 10 mm (with permission from [141]).

Biodegradable polyurethane (PU) is a synthetic polymer with sufficient printability and elasticity, as well as adjustable mechanical properties [141]. Hydrogels comprising gelatin and PU have been found to exhibit adequate printability and modifiable mechanical strength to support MSCs’ proliferation and differentiation [141]. Wu and coworkers combined PU nanoparticles (NPs) with gelatin to develop an elastic planar-/curvilinear bioink. The bioink was laden with three different types of rat cells (i.e., fibroblasts, keratinocytes and EPCs) and bioprinted via an extrusion-based bioprinter to fabricate skin tissue constructs. The latter were placed onto circular or irregular wounds in rats (normal and diabetic models). Increased tissue integration, collagen production, re-epithelialization and angiogenesis were observed 28 days after transplantation (Figure 9) [141]. 

To combine manufacturability with biomimicry, Hao and coworkers (2023) developed a thermo-responsive stepwise multi-crosslinking (i.e., pre-crosslinking via the Michael addition reaction of thiol–acrylate groups before bioprinting, hydrophobic interaction during bioprinting, and “thiol-ene” click reaction of the remaining thiol–acrylate groups under UV after bioprinting) bioink comprising thiolated Pluronic F127 (PF127-SH) and methacrylated hyaluronic acid (HAMA). This strategy was revealed to significantly improve the bioink regarding rheology, printability, mechanical properties, structural integrity, cytocompatibility, etc. Furthermore, MSC-laden printed hydrogels were shown to stimulate wound healing and re-epithelialization via modulation of inflammation and acceleration of collagen deposition and angiogenesis [148].

**Figure 10:**
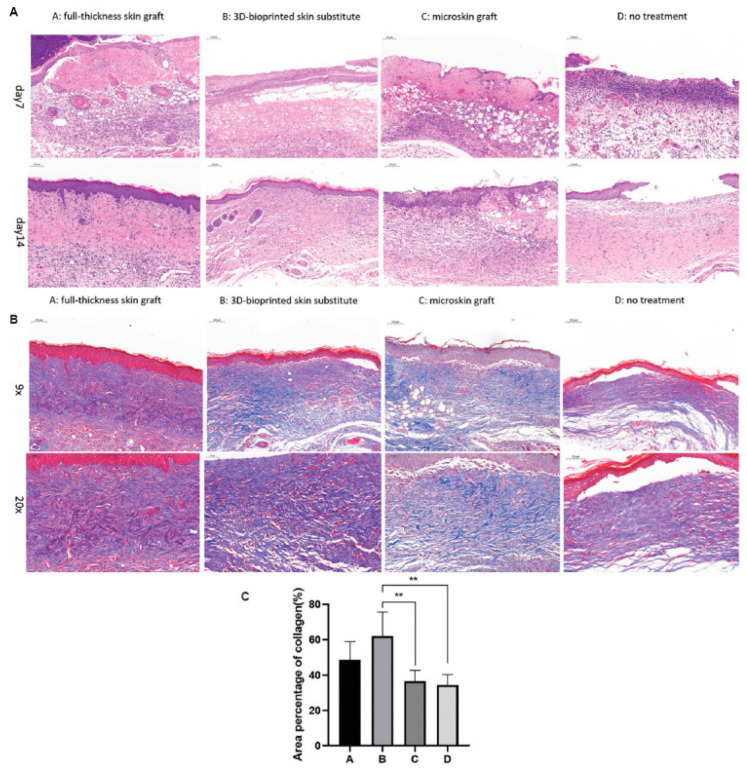
(**A**) Hematoxylin–eosin staining was performed to evaluate the effect of different methods on histology of wound healing on days 7 and 14 after the operation. Scale bar = 100 μm. (**B**) Masson staining was performed to observe the effect of different methods on the production and arrangement of collagen on day 14. Scale bar = 100 μm. (**C**) The area percentage of collagen in each group was quantified. ** *p* < 0.01. [150].

Adipose tissue decellularized extracellular matrix (dECM), which preserves the composition of glycosaminoglycans and proteins (e.g., (micro-)fibrillar collagens, glycoproteins and proteoglycans [149]) in ECM, together with a ranked nanofibrous structure [149], is a thermosensitive biomaterial which could form hydrogels via self-assembly, promote accumulation, proliferation and differentiation of cells, stimulate angiogenesis and endorse skin tissue repair [150]. In a recent study, a skin tissue construct was fabricated via extrusion bioprinting using a novel bioink consisting of dECM pre-gel, GelMA and methacrylated hyaluronic acid (HAMA), and laden with human adipose-derived stem cells (hADSCs). When applied to full-thickness wounds in nude mice, the construct was revealed to exhibit structure stability, speed up wound healing, promote re-epithelialization and collagen deposition/arrangement (Figure 10), reduce inflammatory response, stimulate angiogenesis and enhance blood perfusion [150]. In addition, Sarmin and coworkers demonstrated the compatibility of dECM biomaterials with advanced biofabrication techniques and their potential to be applied as matrices for 3D in vitro models of skin (wounded, diseased, normal) [149].

**Figure 11:**
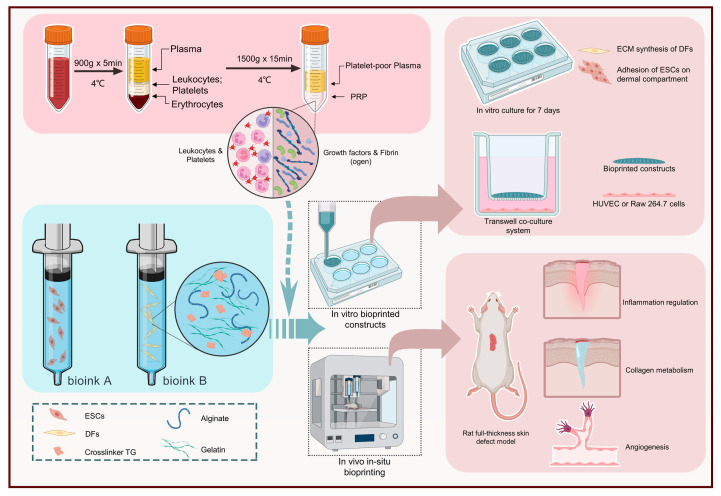
Schematic illustration of bioprinting process using PRP containing multi-component bioink [134].

Platelet-rich plasma (PRP) obtained via centrifugation of whole blood could, at high concentrations, speed up hemostasis through the promotion of thrombosis and coagulation at the wound site. Furthermore, increased concentrations of bioactive agents like growth factors, chemokines, miRNA and immunoglobulins, which exhibit exceptional synergistic effects, could be released by α-particles of PLTs, thus avoiding the disadvantageous application of a single growth factor. Accordingly, PRP has been used in cutaneous wound healing and regeneration/rejuvenation of skin appendages. Additionally, owing to the slow release of bioactive agents, it is anticipated to act for a long time on the wound and achieve the initiation of regenerative signals. Finally, PRP is in alignment with personalized medicine since it is a source of autologous patient-specific growth factors [134]. Zhao and coworkers (2022) developed a hydrogel bioink consisting of alginate–gelatin and PRP (AG-PRPs) for the in situ extrusion-based bioprinting of constructs exhibiting single-layer or double-layer structure and encapsulating dermal fibroblasts (DFs) and epidermal stem cells (ESCs). In situ bioprinting was performed on full-thickness cutaneous wounds in rats by an additive manufacturing system equipped with a robotic arm in order to assess the effect of PRP on the regulation of inflammation, synthesis of collagen, vascularization, etc. (Figure 11) [134].

Skin ECM, with type I and III collagens as its major constituents, is an important part of the skin stem cell niche, supporting cell adhesion, migration, proliferation and differentiation, the essential cellular processes for the formation/regeneration of skin tissue. Equivalents of human skin have been printed using natural biomaterials resembling skin ECM (e.g., collagen type I). However, typically used collagen bioinks exhibit slow gelation under physiological conditions, thus lacking appropriate printability. Additionally, there is a need to further improve the skin equivalent regarding mimicking the complex skin ECM microenvironment. For example, the influence of collagen type III, which has been revealed to be critical for fibrillogenesis of dermal collagen and skin tissue integrity on the formation of skin, is not well understood and has been hardly involved in the fabrication of skin tissue constructs. Yang and coworkers proposed the bioprinting of a full-thickness equivalent of human skin tissue using bioinks based on recombinant human type III collagen [151].

#### 5.1.2. Cells

Fibroblasts and keratinocytes are the frequently used cells in skin TE. Nonetheless, in cases of deep or extensive skin defects, cell extraction from patients is difficult. In this respect, stem cells (SCs) have been favored for skin tissue repair [150]. SCs exhibit desirable properties (e.g., differentiation into different types of tissues and enhanced proliferation) [135]. Initially derived from mesoblasts, adipose tissue-derived stem cells (ADSCs) can effortlessly be obtained in large quantities via minimally invasive methods (e.g., liposuction) in high purity. ADSCs exhibit low immunogenicity and are capable of rapid proliferation in vitro as well as multidirectional differentiation. Additionally, they are characterized by substantial self-renewalability and have been found to endorse wound healing acceleration [150,152]. They are known to have a beneficial role in the entire wound healing process, comprising termination of the inflammation phase, acceleration of both epithelialization and angiogenesis during the proliferative phase and regulation of ECM remodeling. On the other hand, the local delivery of ADSCs to the wound site could result in cell apoptosis due to the generation of mechanical shear force upon injection and the severe microenvironment, thus hindering their direct use in skin TE. To overcome these problems, cells need to be encapsulated in hydrogels where an ambient microenvironment can be maintained prolonging and promoting their survival [147], enhancing their reparative ability, and improving the likelihood of migrating to target tissues [135]. A stem cell microenvironment in living organisms is thought to be heterogeneous because of the stiffness variation between the different skin layers. Cells can sense the stiffness of the substrate and migrate along durotaxis (i.e., stiffness gradient). Accordingly, the application of stiffness gradient around the wound could guide SC infiltration and skin tissue remodeling. Apart from stiffness, hydrogel pore size is considered to be one of the most crucial hydrogel properties since it mediates cell adhesion and paracrine effects and controls stem-cell differentiation. Hence, bioprinting constructs with gradient stiffness and satisfactory pore size might be important for the mediation of ADSCs and could lead to the regeneration of full-thickness skin [147]. 

#### 5.1.3. Bioprinting Methods

Hydrogels can be (bio)printed utilizing various 3D (bio)printing techniques such as extrusion printing, inkjet printing, laser-assisted bioprinting, etc. (Table 6). In extrusion printing, the most commonly used method, the bioink is deposited via the application of pneumatic or mechanical force. This technique is similar to classic hydrogel extrusion from a syringe and is thus readily applied to different hydrogel bioinks. To minimize the imposed shear force on the hydrogel bioink, as well as the required pressure, shear-thinning biomaterials can be used for hydrogel formation. Additionally, monomer solutions of low viscosity can be deposited, which could be crosslinked after printing, or alternatively, materials’ viscosity could be reduced via temperature increase [15]. Inkjet printing comprises the piezoelectric or thermal deposition of small bioink droplets onto a substrate where they spontaneously crosslink and/or fuse leading to the solidification of the printed construct. Laser-assisted bioprinting uses periodic laser excitation for the heating of a donor substrate and the subsequent spatial release of defined bioink droplets adsorbed to the substrate. Digital light processing (DLP) is another method that could aid in overcoming the drawbacks of extrusion or inkjet bioprinting but has not been adequately elucidated. For example, layer-by-layer deposition of cells encapsulated in photocrosslinkable bioinks, with a rapid printing time (1–3 mm/s) and a high resolution (~10 μm) could result in enhanced cell viability in comparison with the other 3D bioprinting methods [146]. Stereolithography is an additional frequently applied method for hydrogel printing involving the sequential curing of horizontal layers of a photocrosslinkable resin into specified geometries. Finally, melt electrowriting applies voltage to generate spatially defined fluid jetting and direct deposition of fibers onto a print platform. Hydrogel bioprinting can be combined with other hydrogel and/or secondary scaffold printing for the generation of composite constructs with enhanced stability and mechanical/biological properties [15]. Hydrogels have been broadly used in bioprinting due to their proven adaptability to 3D printing methods (e.g., extrusion printing, inkjet printing, stereolithography, etc.) [15]. Strikingly, recent bioprinting developments have permitted the fabrication of biomimetic hydrogels promoting wound healing. These hydrogels offer more precise spatial recapitulation of biochemical/biophysical cues which promote tissue repair and augment their therapeutic potential. Accordingly, they have been used for healing acute and chronic wounds [23].

#### 5.1.4. Encapsulation of Bioactive Agents

The encapsulation of bioactive agents and/or drug-loaded nanocarriers (NCs) in hydrogel bioinks can have additional benefits for wound healing. Various NC types have been utilized to increase drug therapeutic efficiency including polymer or lipid-based NPs, liposomes, inorganic NPs, nanohydrogels and nanofibrous structures.

**Figure 12:**
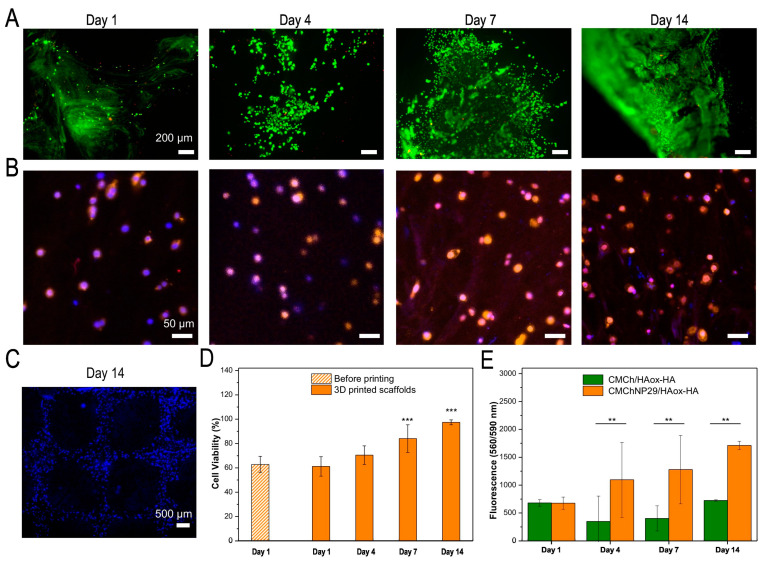
(**A**) Fluorescence images using live–dead staining and (**B**) confocal images of immunostaining of L929 fibroblasts embedded into CMChNP29/HAox-HA bioprinted scaffolds over 14 days of culture. (**C**) Fluorescence image of nuclei staining with DAPI of L929 fibroblasts in a CMChNP29/HAox-HA bioprinted scaffolds after 14 days of culture. (**D**) Quantification of L929 fibroblasts viability in the non-printed hydrogels at 1 day, and in the printed constructs over 14 days for CMChNP29/HAox-HA formulation. ANOVA of the results for printed scaffolds at different time points was performed in comparison with the non-printed sample at significant levels of ** *p* < 0.01 and *** *p* < 0.001. (**E**) Metabolic activity of L929 fibroblasts encapsulated into CMChNP29/HAox-HA and CMCh/HAox-HA bioprinted constructs quantified over time using Alamar Blue staining assay. ANOVA of the results for CMChNP29/HAox-HA sample was performed in comparison with the CMCh/HAox-HA control sample at significant levels of ** *p* < 0.01 and *** *p* < 0.001. (For interpretation of the references to color in this figure legend, the reader is referred to the web version of this article [154]).

Particularly, polymer-based NPs have demonstrated enhanced encapsulation efficiency, thermodynamic stability, drug protection against degradation, topical sustained release, etc. Catechol (i.e., benzene derivative exhibiting anti-inflammatory and antioxidant activity, mitigating inflammation and assisting neovascularization) bearing polymer-based NPs have received a lot of attention due to sustained drug release and reduction of side effects of antimicrobials and various anticancer drugs. In a recent study, Puertas-Bartolome and coworkers (2021) incorporated catechol-bearing polymer NPs encapsulating a model hydrophobic drug in a hydrogel comprising carboxymethyl chitosan and HA for the development of a novel bioink. The bioprinted hydrogel construct was found to exhibit shape fidelity, mechanical stability and uniform distribution of NPs, and support the proliferation of the encapsulated fibroblasts for more than 14 days (Figure 12) [154].

Table 7 presents recent results on the preclinical assessment (i.e., in vivo and in vitro testing, as well as physicochemical characterization) of 3D-bioprinted hydrogels.

## 6. Conclusions

In the last decade, numerous hydrogel constructs have been successfully 3D printed primarily via extrusion bioprinting (~75%) and next by digital light processing (DLP) (~9%). The hydrogel bioinks mostly consisted of sodium alginate (SA) (~29%), gelatin methacrylate (GelMA) or gelatin methacryloil (GelMAl) (~24%) and their mixtures with other natural or synthetic polymers, such as methacrylated hyaluronic acid (HAMA), chitosan (CS), poly (lactic-co-glycolic acid) (PLGA), poly(N-isopropylacrylamide) (PNIPAAm), etc., and/or biological materials like platelet-rich plasma (PRP), decellularized extracellular matrix (dECM), etc., to enhance their physicochemical or biological properties. The developed hydrogel bioinks were mainly crosslinked by photopolymerization (~38%) or ionic crosslinking (~49%) and, in many cases, they were encapsulating bioactive agents (e.g., growth factors, inorganic NPs exhibiting antibacterial properties, antibiotics, DNA, blood derivatives, etc.) (~38%) and/or cells (e.g., fibroblasts, keratinocytes, mesenchymal stem cells (MSCs), human umbilical vein endothelial cells (HUVECs), platelets, etc.) (~28%). The physicochemical characterization of the hydrogel constructs revealed a variety of structures and rheological/mechanical properties along with their ability to release the encapsulated bioactive agents in a controlled manner. Regarding in vitro testing, most of the bioprinted constructs were found to be cytocompatible and to support cell adhesion and proliferation. Finally, in the framework of in vivo testing in normal or diabetic animal models (e.g., SD rats, Wistar rats, nude mice, BALBc mice, New Zealand rabbits, micropigs, etc.), various 3D-bioprinted multifunctional composite hydrogels succeeded in addressing some of the wound healing requirements such as anti-oxidant and anti-inflammatory activity, promotion of healing of full-thickness and/or infected skin defects, granulation tissue formation and growth, re-epithelialization, neovascularization, collagen deposition, minimization of the scar area, etc. On the other hand, despite the promising experimental observations, there are no reported examples of 3D-bioprinted hydrogel constructs that have reached the clinical development phase as potential wound dressings. At this point, it should be mentioned that the design and development of impeccable bioinks are extremely challenging and needs to simultaneously fulfill the requirements for flawless printability, good cell viability, shape fidelity, structure, porosity, degradation, biomimicry, etc., while taking into account the hostile proteolytic wound microenvironment (enriched also with inflammatory cytokines), especially in the chronic wounds, which has a negative effect on cell viability and contributes to the degradation of encapsulated bioactives (e.g., growth factors, pharmaceutics, etc.). Lastly, the lack of consistent protocols for in vivo experiments (e.g., variations in animal models, skin defects, levels of infection) hinders the generation of comparable data and the fabrication of 3D-bioprinted hydrogel constructs for clinical application.

## 7. Future Perspectives

The aforementioned challenges need to be overcome in order to permit 3D-bioprinted hydrogels to have a significant role in the healing of chronic wounds. In this respect, new material combinations should be chosen for bioink development based on systematic high-throughput screening of various natural/synthetic polymers and other biomaterials. In addition, the proper functionalization of the hydrogel construct to combat the proteolytic environment could be considered a crucial approach to promote wound-healing mechanisms and achieve cell survival. Furthermore, novel molecular approaches and genomic tools could be applied to identify and characterize the bacteria residing in the wound bed in order to develop an effective targeted antimicrobial strategy (e.g., controlled delivery of specific antibiotics, biofunctionalization with antimicrobial peptides, etc.). Additionally, the bioprinted hydrogel constructs could be designed in a way to realize a spatiotemporal release of selective bioactive agents according to their beneficial effect (e.g., cell proliferation, angiogenesis, reduction of scarring, etc.). This could be achieved via the direct incorporation of bioactives in the bioink or their encapsulation in nanocarriers incorporated in the bioink to achieve a more sustained release. To achieve the above, advancement in the fields of tissue engineering, bioprinting (e.g., integration of bioprinting with microfluidics to precisely control the structure and composition of hydrogel constructs), cell culture, etc., should be combined with an improved understanding of bioink properties and pathophysiology/biology of wound healing, as well as improved bioprinting protocols and in vivo models better representing human wounds pathology. Furthermore, big data analysis could be utilized to gain information from collected experimental data and machine learning methodologies could assist the acceleration of the hydrogel construct design/development process. Accordingly, bioink selection criteria would require the integration of various scientific fields such as chemistry, engineering, bioinformatics, biology, medical science, etc. Finally, the ultimate goal in the healing of chronic wounds would be in situ bioprinting in a surgical setting enabling real-time printing directly at the patient’s wound with increased anatomical accuracy, thus permitting the creation of customized constructs conforming to the unique wound topology (individualized wound treatment). However, it should be noted that the bioink selection criteria for this approach would be stricter. According to the above, the cooperation of different disciplines and utilization of cutting-edge computational tools could lead to the much-awaited in situ bioprinting of hydrogel constructs for personalized healing of chronic wounds.

## Figures and Tables

**Table 1:** Required characteristics of wound dressings [2,21].

Required Characteristics of Wound Dressings
Absorption of wound exudate;
Permeability of water vapors;
Maintenance of a moist wound environment (at the dressing/wound interface), thus promoting autolytic debridement and removal of dead tissue;
No adherence to the wound surface and easy removal without trauma;
Prevention of microbial (e.g., bacteria, fungi, viruses) transport;
Enhancement of epidermal migration and promotion of angiogenesis and synthesis of connective tissue;
Exchange of gas between wounded skin tissue and the environment;
Mechanical strength to protect the body from further injury;
Biocompatibility/biodegradability and nontoxicity;
Thermal insulation.

**Table 2:** Commercially available film, foam, hydrocolloid, alginate and hydrogel dressings [6,22].

Dressing Characteristics	Activity	Wound Type	Commercial Products
***Film dressings***			
Transparent semi-permeable polyurethane and/or polyacrylate film dressings coated with polyacrylate adhesive. Two-sided wound contact layer with Safetac^®^.	Allow high moisture and vapor transmission. In most cases, provides a barrier to external contaminants (e.g., bacteria, viruses). Absorb low to moderate exudate. Can be used as primary or secondary dressings.	Superficial wounds, closed surgical incisions, minor burns, dry/slightly exuding wounds, cuts and abrasions, donor sites.	Hydrofilm (Hartmann USA, Inc., Rock Hill, SC, USA) [24], Bioclusive™ (KCI, Acelity Inc., San Antonio, TX, USA) [25], Mepore (Mölnlycke, Gothenburg, Sweden) [26], Transeal (Mölnlycke) [27], Tegaderm™ (3M, Maplewood, NJ, USA) [28], OPSITE POST-OP (Smith + Nephew, Watford, UK) [29] Mepitel^®^ (Mölnlycke) [30]
***Foam dressings***			
Polyurethane foam.		Medium to highly exuding, acute and chronic wounds (e.g., diabetic foot ulcers, pressure ulcers II-IV, venous ulcers such as leg ulcers, donor sites, incisions, abrasions).	PermaFoam^®^ (Hartmann) [31]
Hydroresponsive dressing comprising polyurethane foam combined with AquaClear^®^ Technology HydroTac^®^ Comfort is further coated with a waterproof, bacteria-resistant protective film.	Absorbs exudate and at the same time hydrates the wound, thus providing a balanced moist environment. Retains exudate under pressure, thus reducing maceration. Promotes granulation and epithelialization. Alleviates pain on removal.	Wide range of acute and chronic wounds.	HydroTac^®^ (Hartmann) [32] HydroTac^®^ Comfort (Hartmann) [32]
Antimicrobial foam dressing with Safetac technology layer.	Absorbs exudate while maintaining a moist wound environment. The Safetac^®^ technology layer prevents exudate leakage to the surrounding skin, minimizing this way maceration.	Low to medium exuding wounds (e.g., leg/foot ulcers, pressure ulcers). Partial-thickness burns.	Mepilex^®^ Ag (Mölnlycke) [33]
Five-layer polyurethane foam dressing with silicone adhesive.	Minimizes pain during dressing changes. Absorbs and evaporates moisture, thus reducing skin maceration. Suitable for use on fragile skin. Appropriate for use under compression.	Low to highly exuding wounds (e.g., venous leg ulcers, pressure ulcers, arterial ulcers, neuropathic ulcers, surgical wounds, etc.).	Tegaderm™ Silicone Foam Dressings (3M) [34]
Four-layer design including polyurethane foam and acrylate adhesive.	Absorbs and evaporates moisture to maintain an ideal moist wound environment and reduce maceration. Adapts to varying exudate levels. Provides a viral barrier.	Low to highly exuding partial to full-thickness wounds, such as pressure ulcers, diabetic foot ulcers, arterial ulcers, venous leg ulcers, neuropathic ulcers, surgical wounds, abrasions, trauma and skin tears.	Tegaderm™ High-performance Foam Adhesive Dressing [35]
Four-layer design including polyurethane foam.	Absorbs and evaporates moisture to maintain an ideal moist wound environment and reduce maceration. Easy removal. Appropriate for use with compression therapy.	Low to highly exuding partial and full-thickness wounds (e.g., pressure ulcers, ulcers of the lower extremity, donor sites, surgical wounds, abrasions and skin tears). Superficial burns of partial thickness.	Tegaderm™ High-performance Foam Non-adhesive Dressing [36]
Polyurethane foam with an outer layer of vapor-permeable and water/bacteria-resistant polyurethane film.	Absorbs wound exudate and provides a moist wound environment.	Moderate to highly exuding wounds (e.g., pressure ulcers, surgical wounds). First and second-degree burns.	Askina Foam™ (B. Braun, Melsungen, Germany) [37]
Polyurethane foam combined with a highly permeable backing film.	Effective exudate management resulting in a moist wound environment. Suitable for use under compression.	Moderate to highly exuding wounds like leg ulcers and pressure ulcers.	Lyofoam^®^ Max (Mölnlycke) [38]
Hydrocellular foam featuring a low-tack silicone.	Use under compression therapy or other secondary retention to support wound healing via management of wound exudate.	Chronic wounds (e.g., diabetic foot ulcers, venous leg ulcers, pressure injuries). Acute wounds (e.g., burns, surgical wounds, donor sites and skin tears).	ALLEVYN Non-bordered Dressings (Smith + Nephew) [39]
Antimicrobial foam dressing containing silver sulfadiazine which provides sustained anti-microbial action.	Exudate management by secondary intention in order to create a moist wound-healing environment. Reduced risk of infection.	Chronic and acute partial/full-thickness or shallow granulating exuding wounds (e.g., pressure ulcers, diabetic ulcers, venous ulcers, fungating/malignant wounds, burns, surgical wounds, donor sites). Could be used on infected wounds.	ALLEVYN Ag (Smith + Nephew) [40]
Hydrophilic polyurethane foam combined with a waterproof polyurethane film and containing polyhexamethylene biguanide (PHMB).	Prevents granulation tissue formation in the foam, reducing this way trauma during removal. Exudate uptake and prevention of excess fluid, leading to maceration. Appropriate for use under compression.	Moderate to highly exuding chronic and acute wounds, infected or at risk of being infected (e.g., pressure ulcers, leg/foot ulcers, diabetic ulcers, surgical wounds).	TIELLETM PHMB (Acelity Inc., San Antonio, TX, USA) [41] ActivHeal^®^ PHMB [42]
Hydropolymer adhesive dressing.	Exudate handling in order to maintain an appropriate moist wound environment.	Low to moderately exuding wounds (pressure, venous, arterial ulcers, lower extremity ulcers, diabetic ulcers, donor sites). Could be used on infected wounds.	TIELLE^®^ LIQUALOCK (Acelity) [43]
Hydrophilic polyurethane foam combined with an active layer comprising chitosan/alginate, and an outer layer consisting of a semipermeable polyurethane film.	Maintain an optimal moist wound environment promoting wound healing. Hemostatic activity (control of severe bleeding).	Traumatic, postoperative wounds.	Tromboguard^®^ (TRICOMED SA, Lodz, Poland) [44]
Polyurethane pad with high absorption capacity combined with a silicone adhesive layer and an outer waterproof polyurethane film.	Avoidance of sticking to the wound surface, thus facilitating dressing changes. Uptake of exudate and prevention of excess fluid leading to maceration. Maintenance of a moist wound environment aiding wound healing.	Moderate to heavily exuding chronic and acute wounds (e.g., pressure, diabetic, venous and arterial leg ulcers, trauma and surgical wounds, skin abrasions, superficial/partial-thickness burns). Can be used on cavity wounds as a secondary dressing.	ActivHeal^®^ Silicone Foam [45]
***Hydrocolloid dressing***			
Hydrocolloid dressing comprising sodium CMC, gelatin, pectin and adhesive polymers.	Formation of a cohesive gel, creating an occlusive moist wound environment, aiding autolytic debridement and promoting granulation.	Partial and full-thickness low to moderately exudating wounds. Chronic wounds (e.g., stage I-IV pressure ulcers, leg ulcers). Acute wounds (e.g., traumatic wounds such as minor abrasions, lacerations) partial-thickness burns and donor sites.	Granuflex™ (ConvaΤec, London, UK) [46]
Hydrocolloid dressing comprising an elastic/sticky mass encapsulating moisture-absorbing sodium carboxymethylcellulose (CMC) particles and calcium alginate and covered by a semi-permeable polyurethane film.	Formation of a viscous gel that absorbs exudate upon contact with wound. Controlled evaporation through the semi-permeable film. Bacterial barrier.	Low to moderately exuding wounds (e.g., leg ulcers, pressure ulcers, superficial (partial-thickness) burns, surgical wounds, donor sites and skin abrasions).	Confeel^®^ Plus Dressing (Coloplast, Humlebæk, Denmark) [47]
Hydrocolloid dressing comprising an elastic/sticky mass encapsulating moisture-absorbing sodium carboxymethylcellulose (CMC) particles and covered by a semi-permeable polyurethane film.	Formation of a viscous gel that absorbs exudate upon contact with wound. Controlled evaporation through the semi-permeable film. Bacterial barrier.	No to low exuding chronic wounds and superficial acute wounds. Superficial (partial-thickness) burns, surgical wounds, donor sites and skin abrasions.	Confeel^®^ Plus Transparent (Coloplast) [48]
Hydrocolloid dressing consisting of an inner layer that adheres to the skin in the presence of moisture and highly absorbs exudate. Outer water- and bacteria-resisting film.	Protection of wound area and surrounding skin from bacteria/viruses, contaminants, etc. Constantly high moisture vapor transmission, thus reducing the potential maceration	Low to moderately exuding wounds like partial and full-thickness dermal and leg ulcers, superficial wounds, abrasions, donor sites and superficial and partial-thickness burns.	Tegaderm™ Hydrocolloid Dressing (3M) [49]
Hydrocolloid dressing comprising a crosslinked honeycomb matrix of sodium carboxymethylcellulose, gelatin, pectin and adhesive polymers, and an outer waterproof polyurethane film.	Supports moist wound-healing environment and autolytic debridement. Bacterial/viral barrier.	Lightly exuding acute (e.g., minor burns, surgical wounds, abrasions and lacerations) and chronic wounds (e.g., stage I–II pressure ulcers, leg ulcers).	DuoDERM^®^ Extra Thin Dressing (ConvaTec) [50]
Hydrocolloid dressing comprising a crosslinked honeycomb matrix of sodium carboxymethylcellulose, gelatin, pectin and adhesive polymers, and an outer waterproof polyurethane film. Green line indicator to show when the dressing needs to be changed.	Supports moist wound-healing environment and autolytic debridement. Bacterial/viral barrier.	Lightly to moderately exuding chronic (e.g., pressure and leg ulcers) and acute (e.g., minor burns, surgical and traumatic wounds and donor sites) wounds.	DuoDERM^®^ Signal™ Dressing (ConvaTec) [51]
Hydrocolloid dressing featuring a waterproof polyurethane cover film.	Preserves a moist wound environment promoting autolytic debridement while achieving low levels of exudate. Prevents bacterial invasion.	Partial to full-thickness wounds (e.g., venous, arterial, pressure and diabetic ulcers) donor sites, surgical incisions/excisions, and burns (first and second degree).	REPLICARE^®^ Thin Hydrocolloid Dressing (Smith + Nephew) [52]
Hydrocolloid dressing.	Absorbs excess exudate, creates a moist wound environment and assists in autolytic debridement. Insulates the wound and acts as a barrier to external contaminants.	Minimal to moderate exuding partial to full-thickness wounds.	Restore Hydrocolloid Dressing (Hollister, New Albany, OH, USA) [53]
***Alginate dressings***			
Calcium sodium alginate (80% Ca and 20% Na) with a high content of guluronic acid.	Upon contact with wound, calcium ions exchange with sodium ions present in the exudate thus transforming the dry, fibrous dressing to a stable, moist gel, providing this way a moist wound-healing environment. Promotes hemostasis by supporting formation of blood clots.	Moderate to highly exuding chronic (e.g., leg, pressure and diabetic ulcers, fungating lesions) and acute (e.g., donor sites, post-surgical wounds, abrasions and lacerations) wounds. Wounds with negligible bleeding.	Kaltostat^®^ Dressing (ConvaTec) [54]
Calcium alginate containing silver (1.4%).	Upon wound contact, the silver ions are replaced by the sodium ions present in the exudate and are thus released. A gel is formed via absorption of wound exudate, aiding in the maintenance of a moist wound-healing environment. Bacterial barrier.	Moderate to heavily exuding wounds like diabetic foot ulcers, leg ulcers (e.g., venous, arterial ulcers, etc), pressure ulcers, traumatic/surgical wounds and donor sites.	ALGICELL^®^ Ag (Gentell, Yardley, PA, USA) [55]
Alginate dressing containing medical grade Manuka honey.	Creation of a moist wound-healing environment. Antibacterial action through sustained release of Manuka honey.	Various types of wounds including pressure, leg and diabetic ulcers, burns, surgical wounds, graft sites. Suitable for infected wounds.	Algivon and Algivon Plus (Advancis Medical, Hamburg, Germany) [56,57]
Combination of alginate (10%) and collagen (90%).	Maintenance of a physiologically moist microenvironment supporting the formation of granulation tissue, epithelialization and fast wound healing.	Exuding partial and full-thickness wounds, e.g., venous ulcers, pressure injuries, diabetic ulcers, traumatic wounds healing by secondary intention, abrasions, surgical incisions, second-degree burns, donor sites, etc.	Fibracol™ Plus (3M) [58]
Calcium alginate.	Sodium salts from exudate exchange with calcium in the dressing thus forming a hydrophilic gel. Absorption of exudate from the wound while maintaining an optimal moist wound environment. Minor bleeding control via platelet activation caused by released calcium ions.	Moderate to heavily exuding granulating wounds or having areas of slough (e.g., pressure ulcers, venous leg ulcers, arterial ulcers, diabetic ulcers, post-operative surgical wounds, cavity wounds, graft and donor sites, superficial/partial-thickness burns).	ActivHeal^®^ Alginate [59]
Calcium sodium alginate. Calcium sodium alginate with silver.	Creation of a protective gel upon contact with exudate helping this way to maintain a moist wound environment. Protection of periwound skin. Remains intact with saturation. Sustained release of Ag ions. Efficient barrier against bacteria.	Moderate to highly exuding wounds.	Restore™ Calcium Alginate (Hollister) [60] Restore™ Calcium Alginate Dressing with Silver (Hollister) [61]
Non-woven dressing comprising (mannuronic acid) calcium alginate and carboxymethylcellulose.	Release of Ag ions in the presence of exudate. Formation of a soft gel with exudate absorbance which facilitates the maintenance of a moist environment and permits intact removal.	Moderately to heavily exuding partial and full-thickness wounds (e.g., post-surgical and trauma wounds, cavity wounds leg, pressure and diabetic ulcers, burns).	ActivHeal Aquafiber^®^ Ag [62]
***Hydrogel dressings***			
Amorphous gel delivered into the wound through a tube.	Increase in wound moisture level thus boosting moist wound healing via autolytic debridement.	No to low exuding dry/sloughy wounds such as pressure ulcers, leg ulcers, diabetic ulcers, cavity wounds, post-surgical wounds, graft and donor sites, lacerations and abrasions.	ACTIVHEAL^®^ HYDROGEL [63]
Amorphous gel delivered into the wound through a tube.	Provides a moist wound environment and contributes to autolytic debridement. Easy to remove.	Partial to full-thickness wounds dry and minimally exuding wounds. Fill dead space in wounds. Could be applied to infected wounds.	Restore Hydrogel Dressing (Hollister) [64]
Smart, ionic hydrogel dressing (70% water and 30% acrylic polymers) combined with a transparent polyethylene film.	Dynamic capacity for fluid management (i.e., donates moisture to dry wounds and absorbs fluid from exuding wounds). Maintenance of suitable levels of moisture for autolytic debridement and granulation. Pain reduction through cooling of inflamed skin tissue and soothing of irritated tissue.	Dry to moderately exuding chronic and acute wounds (e.g., venous leg ulcers (also under compression), arterial ulcers, diabetic foot ulcers, malignant wounds, moderate burns like those from radiotherapy, skin tears, etc.).	SUPRASORB^®^ G (Lohmann-Rauscher, L&R, Milwaukee, WI, USA) [65]
Hydrogel Sheet Dressing.	Minor exudate absorption. Promotion of moist wound-healing environment. Fluid/bacteria barrier. Cooling and soothing of skin tissue.	Dry to lightly exuding partial and full-thickness wounds (e.g., pressure ulcers, tissue damaged by radiation, minor burns).	AquaDerm™ (DermaRite, North Bergen, NJ, USA) [66]
Hydrogel Sheet Dressing (3 mm thick) consisting of natural and synthetic polymers like polyvinyl pyrrolidone, polyethylene glycol and agar and containing 90% water.	Promotion of rapid healing via maintaining an optimal moist wound environment aiding autolytic debridement. Positive effect on granulation and epidermization. Prevention of bacteria invasion.	First-, second- and third-degree burns, ulcers and bedsores.	Neoheal^®^ KIKGEL [67]
Clear hydrogel dressing containing modified carboxymethyl cellulose, propylene glycol and water [2].	Promotion of wound healing through creation of a moist wound environment. Promotion of autolytic debridement. Bacteriostic properties protect the wound against external contamination and the risk of infection. Cooling effect minimizing patient pain.	Shallow, undermined, and deep wounds, granulating cavity wounds, excoriated skin and radiation burns.	INTRASITE◊ GEL (Smith + Nephew) [68]
Transparent, amorphous hydrogel containing sodium alginate.	Creates a moist wound-healing environment, which assists with natural autolytic debridement by donating moisture to the wound bed whilst the alginate component enhances its absorptive capabilities. Soften and hydrate eschar by facilitating rehydration of the wound.	Necrotic and sloughy wounds.	Nu-Gel™ (3M™) [69]

**Table 3:** Cell-based dressings [4].

Category	Commercial Product
Amniotic/placental membranes	Dehydrated EpiFix^®^ (MiMedx Group, Marietta, GA, USA) Cryopreserved Grafix* (Osiris Therapeutics, Inc., Columbia, SC, USA)
Bovine type I collagen seeded with human allogeneic skin cells	OrCel^®^ (Ortec International, New York, NY, USA) Apligraf^®^ (Organogenesis Inc., Canton, OH, USA) Polyglactin mesh scaffold Dermagraft^®^ (Organogenesis Inc.)
Allogeneic fibrin patches containing leukocytes and platelets	LeucoPatch^®^ (Reapplix, Birkerød, Denmark)

**Table 4:** Preclinical evaluation of 3D-printed hydrogels as wound dressings.

Materials	Crosslinking Method	3D Printing Method	Bioactive Agents	Dressing	Animal Model/Wound Type (In Vivo) or Cell Type (In Vitro)	Outcome
**In vivo**						
FSA [79]	Ionic	Extrusion	DNA, biomineralized silica	Bioactive hydrogel	Normal and diabetic mice Male BALB/c mice (8 weeks) Male C57BL/6 mice (8 weeks)/full-thickness wounds	Absence of mechanical deformations due to AI-based 3D printing Increased blood and exudate absorption Enhanced ROS scavenging, anti-inflammatory activity and angiogenesis resulting in accelerated wound healing (both acute and diabetic)
SA, CHSMA [80]	Ionic and photocrosslinking	Extrusion	Acrylylated VEGF	Double-crosslinked angiogenic hydrogel	T1DM/full-thickness wound	Prolonged release of VEGF due to chemical coupling Enhanced mechanical properties due to double crosslinking Better fit at wound site Extensive neovascularization and formation of mature epithelium
SA, GG, PDA NPs [13]	Ionic			SA-GG@PDA hydrogel	Female C57BL/6 mice (3–5 weeks) injected with B16F10 melanoma cells (5 × 10^5^ cells)/full-thickness wound (Ø 10 mm)	Promotion of post-surgery wound healing
SA, CMC [98]	Ionic	Pneumatic extrusion		SA/CMC hydrogel scaffolds with two-tier structures	New Zealand rabbits/full-thickness wound (Ø 2.5 cm)	Wound healing comparable to autologous skin graft
PLGA (upper layer), SA (lower layer) [81]	Ionic			Bilayer membrane scaffold	Female SD rats (10 weeks)/10 mm biopsy punch wounds	Prevention of bacterial invasion in vitro Preservation of hydrogel humidity Promotion of inflammation, neovascularization and collagen I/III deposition in vivo Acceleration of wound healing
SA-DA, gelatin [83]	Ionic	Co-axial		Hollow channeled hydrogel scaffold	BALB/c mice/infected wound (Ø 8 mm)	Exceptional antibacterial activity against *E. coli* and *S. aureus* Promotion of angiogenesis Acceleration of wound healing
Gelatin [14]		Extrusion	MH		Chicken embryo	Antibacterial activity and boost of the proliferation of human dermal fibroblasts and epidermal keratinocytes Lack of irritation and promotion of angiogenesis
Gelatin, PRP, SA, CS [93]	Ionic dual crosslinking	Coaxial microfluidic bioprinting		Βioactive shell-core fibrous hydrogels	SD rats with Type 2 diabetes/full-thickness skin wounds	Sustained release of GFs Effective inflammation reduction Promotion of granulation tissue growth and angiogenesis Excellent candidate for diabetic foot ulcer treatment
GelMA [72]	Photocrosslinking		VEGF mimicking peptide	Hydrogel patches	Micropigs (30–35 kg)/full-thickness excision wound	Acceleration of wound healing through promotion of collagen deposition and angiogenesis
GelMA-alginate (bottom layer) PEGDA-alginate (top layer) [99]	Photocrosslinking and ionic	Extrusion	Au-decorated BTO nanocubes, i.e., piezoelectric nanomaterials (top layer) VEGF (bottom layer)	Janus hydrogel patch	SD rats/full-thickness excision wound (infected dorsal wounds)	Significantly enhanced antibacterial activity due to increased ROS production Effective alleviation of infection and promotion of angiogenesis Promotion of tissue regeneration via sustained growth factor release
GelMa, PEDOT, PSS [100]	Photopolymerization		Electrical stimulation	Electro-conductive hydrogels with grooved topography	New Zealand rabbits/full-thickness wound	Increased cell proliferation, directional migration and orientation Regeneration of full-thickness skin
GelMAl [96]	Photocrosslinking		VEGF decorated photoactive and antibacterial t-ZnO MPs	Hydrogel with t-ZnO-VEGF	Male SKH-1 hairless mice (6 weeks)/circle defects (9 mm)	Light-induced controlled release of VEGF The hydrogel patches exhibited low cytotoxicity and enhanced angiogenic and antibacterial properties in vitro The 3D printed hydrogel with t-ZnO-VEGF exhibited increased anti-inflammatory activity, induced angiogenesis and cell proliferation, and achieved wound healing in vivo
GelMA, HAMA, PRP [101]	Photocrosslinking	DLP		FDPBH	Diabetic male SD rats (4 weeks)	Modulation of cell behavior Facilitation of wound healing
Dopamine (coating), Gelatin/SA (core), PCL (shell) [102]	Ionic	Extrusion		Dopamine-coated core–shell (gelatin/SA/PCL) hydrogel	Balb/C female mice/full-thickness wound	Superior wound healing performance Minimization of scar area
SS, GelMA [103]	Photocrosslinking	Extrusion		Hybrid IPN SS/GelMA hydrogel	Female SD rats	Induced proliferation of L929, HSF and HaCaT Cells No significant inflammatory reaction in vivo Promising candidate for wound healing applications
SF, gelatin [104]	Photocrosslinking	Extrusion	MB-loaded UiO-66(Ce) NPs	SF/gelatin hydrogel loaded with MB@UiO-66(Ce) NPs	Kunming mice (8 weeks)/ full-thickness infected skin defect (Ø 5 mm)	Promotion of fibroblasts’ migration and proliferation Acceleration of wound repair Reduction of viability of *S. aureus* and *E. coli* with laser treatment
CS [87]	Thermal gelation	DIW extrusion		Green CS hydrogels of increased strength	Male SD rats/full-thickness skin wounds (Ø 15 mm)	Biocompatible hydrogels with enhanced mechanical properties Decreased expression of pro-inflammatory factors Increased expression of vascular and anti-inflammatory factors
HA/CS (top layer), HA/CS and PLA nanofibrous MS (bottom layer) [105]	Chemical	Extrusion	ZNP or D-ZNP	Hydrogel construct	Diabetic male Wistar rats (225 ± 25 g)/full-thickness excision wounds (Ø 1.5 cm)	Treatment of full-thickness diabetic wounds infected with S. aureus
CS, SA, PVA [106]		Coaxial biological 3D printing technology	MMP inhibitor, indomethacin	Dressings based on core–shell hydrogel fibers	Type 2 diabetic SD rats/wounds of sufficient depth to reach fascia	Exceptional absorption and retention of moisture Excellent antibacterial activity (up to 98%) Promotion of L929 cells growth, proliferation, and adhesion. Reduction of inflammation, promotion of granulation tissue/capillaries growth, and formation of highly oriented collagen fiber networks
CNCs, CS-MA [75]	Photocrosslinking	Extrusion	VEGF, gentamicin, silver NPs	Mesh-like hydrogel 3D printed on Tegaderm	Female C57BL/6J mice (6–8 weeks)/full-thickness excision wound	Improved granulation tissue formation in comparison with the control (Tegaderm)
CMC, GMA, ε- PL [90]	Photocrosslinking	Extrusion		Bionic antibacterial and antioxidant hydrogels	SD rats/full-thickness infected skin wound	Adhesion to skin High inhibitory effect on *S. aureus* and *E. coli* Removal of excessive ROS Protection of fibroblasts Increased expression of VEGF and CD31, and accelerated wound healing in comparison with Tegaderm™ film
PAM, HPMC [92]	Silver–ethylene interaction	FDM 3D printing (for the PLA template)	Silver NPs	Superporous antibacterial hydrogels	SD rats/full-thickness wound (1 × 1 cm)	Low release rate of silver NPs form hydrogels resulting in balanced antibacterial activity and cytocompatibility Decreased risk of dressing detachment Promotion of infected wound healing and formation of smooth tissue
RHCMA, HAMA [107]	Photocrosslinking		AgNCs		Diabetic model	Antimicrobial activity against *S. aureus* and *P. aeruginosa* Promotion of fibroblasts’ migration and proliferation Regeneration of tissue and deposition of collagen in diabetic wounds
OMS [108]	Ionic	Green hot extrusion			Male C57BL/6 mice (6–8 weeks)/skin wounds (5 × 5 mm)	Exceptional hemostatic (only 65 s) and wound healing activity Promotion of EGF secretion as well as VEGF expression
BSA and AV gel [109]	Thermal	Extrusion			Diabetic rats/full-thickness open-excision wound	Antioxidant/antibacterial properties
NIPAAm, SA [94]	Ionic and photocrosslinking	Microfluidic-aided 3D printing	MXene, VEGF	Dynamically responsive channeled hydrogel scaffolds	Male C57BL/6 mice (22–24 g)	NIR-responsive (reversible) shrinkage and swelling activity Enhancement of regenerative capacity in vitro (cell proliferation/ migration, pro-angiogenicity) Increased survival rate of skin flap through promotion of angiogenesis, reduction of apoptosis and decrease in inflammation
PEGDA [110]	Photocrosslinking		GaM	Hydrogel dressing with hierarchical porosity	C57BL/6 mice (2 months)/splinted-wound	Prominent reduction of bacterial load in the wound without affecting wound closure rate
PVDF, SA [111]	Ionic		ZnO NPs	Piezoelectric hydrogel	Adult female SD rats/ full-skin wound	Biocompatible dressing with antimicrobial and wound-healing properties Effective prevention of scar formation
EW [112]	NaOH, DMEM (secondary crosslinking)	Extrusion	ASCs L929 Fbs		C57 mice/full-thickness skin defect (Ø 1 cm)	Facilitation of wound healing via promotion of angiogenesis and rearrangement of collagen
ECM, SA, gelatin [95]	Ionic and chemical	Extrusion			Male BALB/c, (25–30 g)/full-thickness circle wound (area~100 mm^2^)	Enhanced formation of granulation tissue, re-epithelialization and angiogenesis
ECMMA [113]	Photocrosslinking	Extrusion	Cu-EGCG capsules	ECMMA/Cu-EGCG dermal scaffold	Male SD rats (200−250 g)/a circular full-thickness skin wound (Ø 20 mm)	Alleviation of pathological scarring by combining the ECMMA/Cu-EGCG dermal scaffold with split-thickness skin graft transplantation
Casein [114]	Photocrosslinking with white light	DLP			Male C57BL/6 N mice (8 weeks, 20 ± 2.5 g)	Exceptional hemostasis Appropriate for first-aid wound treatment Facilitate healing of post-traumatic wounds
**Ex vivo**						
SA [115]	Ionic		t-ZnO		Human skin explants	Ideal carrier for protein administration Antibacterial activity against *S. aureus* Compatibility with skin
**In vitro**						
SA [116]	Ionic	Extrusion	ZnO NPs	SA hydrogel with embedded ZnO NPs	Mitomycin-C-treated STO fibroblasts	Decrease in bacterial growth in comparison with SA hydrogels No adverse effect on cell viability by the addition of ZnO NPs to the SA gels
SA [82]	Ionic	Extrusion	HZJ bacteriophage that targets DH5α *E. coli*	Bacteriophage-based SA hydrogel	L929 fibroblasts	80% of encapsulated phages remained lytic Slow release of embedded phages Antibacterial activity sustained for ≥24 h Promoted the proliferation of L929 fibroblasts
SA, HA [117]	CaCO_3_, GDL	Extrusion-based 3D printing on a stainless steel cold (−14 °C) Peltier cell	LD derivatives	Self-crosslinked SA-HA hydrogels functionalized with LD derivatives	Primary human skin fibroblasts	Enhanced proliferation and migration of fibroblasts
SA/CNCs and SA/T-CNF [118]	Ionic	Extrusion		Hybrid hydrogels	NIH/3T3 mouse fibroblasts	CNF exhibited superior printability, rheological/mechanical properties and morphological structure, chemical and shape fidelity, and biocompatibility
SA, gelatin [119]	Ionic	Extrusion			Keratinocytes (HaCaT)	Good structural and mechanical characteristics Keratinocytes’ aggregation into spheroids
ADA-GEL [84]	Ionic		ASX and BBG MPs		NIH 3T3 fibroblasts Keratinocytes (HaCaT)	Delayed hydrogel degradation due to hydrogen bonding of incorporated agents with the polymer chains Promotion of cell adhesion and proliferation as well as expression of VEGF Promotion of keratinocyte migration
SA, MC [120]	Ionic	Extrusion	MH, eucalyptus essential oil, AV gel	SA-MC hydrogels	HDFs	Biocompatible hydrogels inducing cell growth Antimicrobial activity against Gram-positive and negative bacteria
SA, XG [121]	Ionic	Extrusion	CLV		Raw 264.7 macrophage-like cells	Sustained release of CLV Antimicrobial activity against *S. aureus* and *E. coli* Possible anti-inflammatory activity
GelMA [86]	Photocrosslinking	Extrusion	CaS extracts with various concentrations of Si ions		-	Increased adhesion and proliferation of HDFs Enhanced expression of biomarkers related to ECM remodeling Increased HDF activity because of gradual release of Si ions
Gelatin, dECM, QCS [122]	Chemical	Extrusion		Gelatin–dECM–QCS composite scaffolds	L929 cells (5 × 10^5^ cells)	Antimicrobial activity against *E. coli* and *S. aureus* Possible exosome carrier Promotion of fibroblasts’ adhesion, proliferation and migration
Gelatin, SA, QCS, dopamine [123]	EDC/NHS and ionic	Extrusion		Double-crosslinked gelatin/SA/ dopamine/ QCS hydrogel	L929 fibroblasts	Considerable tensile strength and degree of swelling Antibacterial activity against *E. coli* and *S. aureus* High biocompatibility
T-CNF, GelMA [89]	Ionic and photocrosslinking	Extrusion		T-CNF/GelMA hydrogel	3T3 fibroblasts	Tunable hydrogel mechanical strength Promotion of fibroblast proliferation
CS, SA [124]	Ionic	Extrusion	SSD	CS/SA hydrogels	Human fibroblasts	Decrease in SSD cytotoxicity via fine tuning of its release Strong antimicrobial activity against *P. aeruginosa* and *S. aureus*
CS or SA [125]	Ionic	Extrusion	Ag NPs and TiO_2_		Human fibroblasts	Antimicrobial activity against *S. aureus* and *P. aeruginosa*
CS, P(KA), 4-PEG [73]	Chemical (P(KA)/4-PEG MPs) Photocrosslinking (CS)-P(KA)/4-PEG	Extrusion	-		NIH 3T3 mouse fibroblast cells	The composite polypeptide-based microgel inks were shown to promote adhesion and spreading of NIH 3T3 mouse fibroblast cells for 4 days
Calcium D-gluconate monohydrate, SA [126]	Ionic	Printing in a vertical orientation	Bifunctional NMs: PMO functionalized with Dex and PDL	3D step-gradient nanocomposite hydrogels comprising different hydrogel layers with increasing amounts of bifunctional NMs	Primary dermal fibroblasts	The bifunctional NMs were shown to improve cell viability and promote cell migration in the vertical direction (XZ) of the step-gradient nanocomposite hydrogels Cell migration was increased by increasing the concentration of the functional NMs
CNFs [72]	Double crosslinking: ionic and chemical BDDE			Nanocellulose hydrogel	HDFs	Tunable hydrogel mechanical strength Promotion of cell proliferation Enhanced cell proliferation with hydrogel rigidity
PCL, SS (epidermis layer) CS, SA (dermis layer) [127]	Ionic	Electrospinning Extrusion		3D skin asymmetric construct		Non-cytotoxic for NHDFs Confirmed ability of epidermis layer to act as a barrier to the colonization of microorganisms at the wound site Good wettability, mechanical and antimicrobial properties
PNIPAAm, SA, MC [91]	Photocrosslinking and ionic	Temperature-controlled pneumatic-based extrusion printing	Octeisept^®^	Thermoresponsive hydrogel with antimicrobial properties	Fibroblasts L929	Temperature-controlled hydrogel shape, swelling and drug release Enhanced antibacterial/ antifungal properties Non-cytotoxic for fibroblasts
**Physicochemical characterization**						
SA, MC and SA, MC, Laponite (core/shell) [128]		Core/shell extrusion-based printing	Antibiotics (clindamycin vancomycin, gentamicin)	Antibiotic-loaded core/shell scaffolds		Maintenance of hydrogel architecture Fine tuning of antibiotic release
SA, CNC (shell), HEC (core) [129]		Coaxial	TO loaded pegylated liposomes (core), TO (shell)	Composite core/shell scaffolds		Successful combination of liposomes and hydrogels Antibacterial activity against *S. aureus* and *P. aeruginosa*
CS, PEC [130]	Physical (hydrogen bonding)	Extrusion	LDC	Lyophilized hydrogel with sponge-like morphology		Absorption of exudates and maintenance of a moist environment suitable for wound healing Skin adhesion (porcine skin) LDC release over 6 h
CS, PEC [88]	Physical crosslinking			CS/PEC hydrogels		Enhanced degree of swelling, adequate for wound exudate absorption
CSMeA [97]	Photocrosslinking		LIDHCl, LVX	Drug-loaded hydrogels of customizable shape		Formation of hydrogels that can be customized regarding architecture and composition Fine tuning of drug loading and release
XG, LBG, KG, CA [131]	Physical crosslinking		BITC			Enhanced antibacterial activity Good antimicrobial activity against methicillin-resistant *S. aureus* when simulating porcine skin infection after burn
Avidin/T-CNF, SA [76]	Ionic	Microdispensing		Biofunctionalized T-CNF/SA hydrogel		Good tissue compatibility Water and moist absorption Possibility to immobilize bioactive agents through biotin–avidin coupling
Hep-SH, HA-GM [132]	Photocrosslinking	DLP	GFs	Hydrogels of complex geometry		User-defined hydrogel structure controlling the release of GFs
HEMA [78]	Photocrosslinking		pH indicator	Auxetic wound dressing		Adherence to joint motion Could aid in monitoring changes in pH at and close to the wound site

3D: three dimensional; FSA: functionalized sodium alginate; DNA: deoxyribonucleic acid; AI: artificial intelligence; ROS: reactive oxygen species; SA: sodium alginate; CHSMA: chondroitin sulfate methacryloil; VEGF: vascular endothelial growth factor; T1DM: Type 1 diabetes mice; GG: gellan gum; PDA: polydopamine; NPs: nanoparticles; CMC: carboxymethyl cellulose; PLGA: poly (lactic-co-glycolic acid); SD: Sprague-Dawley; SA-DA: dopamine modified alginate; *S. aureus*: *Staphylococcus aureus*; MH: Manuka honey; PRP: platelet-rich plasma; CS: chitosan; GF: growth factor; GelMA: gelatin methacrylate; PEGDA: poly(ethylene glycol) diacrylate; BTO: tetragonal barium titanates; PEDOT: poly(3,4-ethylenediox-ythiophene); PSS: polystyrene sulfonate; GelMAl: gelatin methacryloil; t-ZnO: tetrapodal zinc oxide; MPs: microparticles; HAMA: methacrylated hyaluronic acid; DLP: digital light processing; FDPBH: PRP-loaded bionic-structured hydrogel; PCL: polycaprolactone; SS: silk sericin; IPN: interpenetrating network; HaCaTs: human epidermal keratinocytes; SF: silk fibroin; MB: methylene blue; *E. coli*: *Escherichia coli*; DIW: direct ink writing; HA: hyaluronic acid; PLA: poly(lactic acid); MS: microspheres; ZNP: nano zinc oxide; D-ZNP: didecyldimethylammonium bromide-treated ZNP; PVA: polyvinyl alcohol; MMP: matrix metalloproteinase; CNCs: cellulose nanocrystals; CS-MA: chitosan methacrylamide; GMA: glycidyl methacrylate; ε-PL: ε-polylysine; PAM: polyacrylamide; HPMC: hydroxypropyl methylcellulose; FDM: fused deposition modeling; RHCMA: methacrylated recombinant human collagen; AgNCs: silver nanoclusters; *P. aeruginosa: Pseudomonas aeruginosa*; OMS: oxidized maize starch; EGF: epidermal growth factor; BSA: bovine serum albumin; AV: aloe vera; NIPAAm: N-isopropylacrylamide; MXene: titanium carbide; PEGDA: poly(ethylene glycol)-diacrylate; GaM: gallium maltolate; PVDF: polyvinylidene fluoride; ZnO: zinc oxide; EW: egg white; DMEM: Dulbelcco’s modified Eagle’s medium; ASCs: adipose-derived stem cells; ECM: extracellular matrix; ECMMA: methacrylated decellularized extracellular matrix; Cu-EGCG: copper epigallocatechin gallate; CaCO_3_: calcium carbonate; GDL: δ-glucono lactone; LD: *Lactobacillus bulgaricus*; CNCs: cellulose nanocrystals; T-CNF: TEMPO-oxidized cellulose nanofibers; TEMPO: 2,2,6,6-tetramethylpiperidine-1-oxyl radical; ADA-GEL: alginate dialdehyde–gelatin; ASX: astaxanthine; BBG: borate bioactive glass; MC: methyl cellulose; HDFs: human dermal fibroblasts; XG: xanthan gum; CLV: clove essential oil; CaS: calcium silicate; dECM: decellularized extracellular matrix; QCS: quaternized chitosan; EDC: 3-(3-Dimethylaminopropyl)-1-ethylcarbodiimide hydrochloride; NHS: N-hydroxysuccinimide; SSD: silver sulfadiazine; TiO_2_: titanium dioxide; P(KA): poly(L-lysine-ran-L-alanine); 4-PEG: four-arm poly(ethylene glycol); NMs: nanomaterials; PMO: periodic mesoporous organosilica; Dex: dexamethasone; PDL: poly-D-lysine; CNFs: cellulose nanofibers; BDDE: 1,4-butanediol diglycidyl ether; NHDFs: normal human dermal fibroblasts; PNIPAAm: Poly(N-isopropylacrylamide); HEC: hydroxyethyl cellulose; TO: thyme oil; PEC: pectin; LDC: lidocaine; CSMeA: chitosan methacrylate; LIDHCl: lidocaine hydrochloride; LVX: levofloxacin; LBG: locust bean gum; KG: konjac glucomannan; CA: carrageenan; BITC: benzyl isothiocyanate; Hep-SH: thiolated heparin; HA-GM: glycidyl metharylated hyaluronic acid; HEMA: 2-hydroxyethyl methacrylate.

**Table 5:** Bioprinting technical challenges [8].

Bioprinting Technical Challenges
Production of bioinks with good biocompatibility and at the same time proper rheological/mechanical properties permitting the culture of various cell types;
Enhanced printing resolution is necessary for the construction of inner microarchitectures;
Optimization of printing parameters (e.g., dispensing pressure, extrusion speed, nozzle diameter, droplet size, bioink viscosity, printing time, substrate thickness, laser energy, cell differentiation, etc.);
Development of bioinks conforming to native skin tissue;
Formation of functional vascularized constructs;
Post-printing control of bioprinted construct regarding cellular dynamics, deformation/stiffness.

**Table 6:** Comparison of 3D bioprinting methods [4,15,146,153].

Characteristics	Bioprinting Method			
	*Extrusion*	*Inkjet*	*Laser Assisted*	*Digital Light* *Processing*
***Bioink viscosity (mPa s^−1^)***	30–6 × 10^7^	3.5–12	1–300	
***Crosslinking method***	Photopolymerization, chemical, shear thinning, temperature	Photopolymerization, chemical	Photopolymerization, chemical	Photopolymerization
***Bioprinter speed***	Slow	Fast	Medium fast	Fast
***Droplet size***				
***Cell density (cells/mL)***	High (cell spheroids)	Low (<10^6^)	Medium (10^8^)	High
***Cell viability (%)***	40–80	>85	>95	High
	**Advantages**			
	Easy setup and printing process Compatible with the majority of hydrogels Printing of highly viscous bioinks Printing of high cell densities Accurate control of printheads and bioink conditions Reasonable cost Potentially easy scale up	Fast printing Low cost Accurate control of printheads and bioink conditions Availability	Relatively fast printing High resolution Precise fabrication (i.e., horizontal patterning of biomolecules/cells) Cytocompatible printing conditions because of laser absorption by donor substrate	Fast printing High accuracy High resolution High cell viability
	**Disadvantages**			
	Lower resolution in comparison with other bioprinting methods Shear stress on bioinks Possibility for clogging of printheads Difficult to print overhanging parts Cell structure distortion	Necessity for more specific bioinks of low viscosity Possibility for clogging of printheads Piezoelectric/thermal conditions could induce cytotoxicity Lack of exact droplet size	High cost Necessity for specific bioinks that can be adsorbed to donor substrate Printed constructs of limited scale Potential requirement for UV light of high intensity, and long post-processing Some inks may lack biocompatibility	

**Table 7:** Preclinical evaluation of 3D-bioprinted hydrogels as wound dressings.

Materials	Crosslinking Method	3D Printing Method	Bioactive Agents	Cells	Dressing	Animal Model /Wound Type	Outcome
**In Vivo**							
GelMAl [152]	Photocrosslinking	Extrusion	CUR	ADSCs		nu/nu athymic nude mice induced with diabetes/full-skin defect	Improved antioxidant activity Alleviation of ADSC apoptosis Acceleration of wound healing in diabetic mice
GelMAl/rhCol3 [151]	Photocrosslinking	Extrusion		HDFs (1 × 10^6^/mL) HaCaTs (2 × 10^5^/cm^2^) seeded on the upper surface of the printed skin construct		SD rats (8 weeks)/full-thickness wounds (Ø 5 mm)	Enhancement of keratinocyte proliferation in the epidermal layers Accelerated wound healing in vivo
GelMAm, HAMA, fibrin (bioink), PCL frame, gelatin lower layer [155]	Photocrosslinking and polymerization with thrombin	Extrusion		HUVECs and NHDFs		Nude mice (6–8 wks)/full-thickness wounds (Ø 10 mm)	Printability, biocompatibility, optimal mechanical strength Promotion of neovascularization and wound repair via creation of a patterned surface, enhancement of blood vessel connections, deposition of collagen, etc.
GAH [147]	Ionic	Extrusion		ADSCs	GSH	Male C57BL/6 mice/full-thickness skin defects (Ø 10 mm)	Promotion of angiogenesis and healing due to enhancement of paracrine secretion of cells in the GSH
Gelatin, SA [156]	Ionic	Extrusion		NHDFs, HMVECs (bottom layer) and NHEKs (top layer)		Nude mice/full-thickness wound	Participation of cells in the healing process Improvement in wound contraction by ~10% in comparison with control The newly formed skin appeared similar to the normal skin and exhibited enhanced degree of angiogenesis
GH/AT [138]	Photocrosslinking and ionic	Extrusion		NIH 3 T3 and hUC-MSCs		ICR male mice/full-thickness skin defect	Homogeneous distribution of cells Significant cell survival Facilitation of wound healing via regulation of inflammation, promotion of collagen deposition, acceleration of angiogenesis and formation of skin appendages
PU, gelatin [141]	Ionic	Extrusion		Fibroblasts, keratinocytes and EPCs	Planar-/curvilinear bio-printable hydrogel	Normal, DM rats/full-thickness circular (Ø 1.5 cm) and irregular wounds	Curvilinear bioprinting of irregular tissue-engineered skin Complete repair of wounded skin after 28 days
γ-PGA-GMA, γ-PGA-SH, RGDC [157]	Photocrosslinking	DLP		HUVEC vegf165+	HUVECvegf165+-laden microporous hydrogel	Diabetic male SD rats (7–8 weeks, ~220 g)/full-thickness skin defects (Ø 10 mm)	Acceleration of diabetic wound healing in rats
PF127-SH and HAMA [148]	Michael addition, self-assembly and photocrosslinking	Extrusion		MSCs		Male ICR mice (~20 g)/ Full-thickness skin defects (Ø 7 mm)	Promotion of wound healing and re-epithelialization via modulation of inflammation and acceleration of collagen deposition and angiogenesis
dECM pre-gel, GelMA and HAMA [150]	Photocrosslinking	Extrusion		hADSCs		Nude mice/full-thickness skin wound	Accelerated wound healing Improved healing quality via promotion of angiogenesis
PRP, AG [134]	Ionic and enzymatic	In situ extrusion		DFs and ESCs		Male SD rats/full-thickness cutaneous defects (Ø 15 mm)	Facilitation of ECM synthesis and macrophage polarization Acceleration of wound closure, modulation of inflammation and initiation of angiogenesis
**In Vitro**							
SF-GMA, gelatin-GMA [146]	Photocrosslinking	DLP		HUVEC, HDF and NHEK	Artificial skin	Printed scars of various shapes (skin wound model)	Appropriate matrix for cell proliferation and vascularization Wound healing was observed by applying EGF to the skin wound model. Novel platform for skin TE and wound-healing applications
FSA [79]	Ionic	Extrusion	DNA, biomineralized silica	NIH3T3/GFP fibroblasts	Bioactive cell-laden hydrogel		Uniform distribution of fibroblasts. Successful maintenance of cell viability Enhancement of cell proliferation in the presence of DNA and by increasing silica content
SA [158]	Ionic	Extrusion	CEpCUR	HaCaTs	SA:CEpCUR living construct		Good stability Increased cell viability (~90%) at 7 days post-bioprinting
SA, fibrinogen [159]	Ionic, enzymatic polymerization with thrombin	Extrusion		NHDFs, HaCaTs (seeded on top of the bioprinted construct)	Skin equivalent		Similar architecture with native skin
SA, gelatin, collagen [133]	Ionic	Extrusion		Fibroblasts (2 × 10^6^ cells/mL)	Full-thickness hydrogel with gradient pore structure		Promotion of oxygen/nutrient perfusion Metabolic waste excretion Acceleration of wound healing and reduction of wound contraction
SA, gelatin and DCEL [136]	Ionic	Pneumatic-based extrusion		Primary adult fibroblasts and primary epidermal keratinocytes			Exceptional viability of fibroblasts and epidermal keratinocytes Enhanced collagen synthesis Excellent skin-specific marker and biomimetic tissue histology
MC, SA [143]	Ionic	Extrusion	gallium (Ga^+3^)	Foreskin fibroblast cells			Exceptional antibacterial activity towards *S. aureus* and *P. aeruginosa* Excellent biocompatibility
pDAM2, SA [160]		Extrusion	blood derivatives (platelets, plasma growth factors)	Platelets, human dermal cells	Interactive composite hydrogels		Dynamic up-regulation of healing proteins exhibiting a corrective role in stagnant wounds Stimulation of cell proliferation
Gelatin, XG [85]	Chemical (GTA)	Extrusion		HDFs and human keratinocytes	Hybrid composite hydrogels		In vitro growth of HDFs and human keratinocytes Gradual replacement of the hydrogel with secreted ECM
CMCh, HA [154]	Chemical (formation of Schiff-bases)	Reactive mixing extrusion	polymer NPs containing catechol	HDFs			Controlled release of catechol-containing NPs to the wound Enhanced viability and proliferation of fibroblasts
XRU-MA [160]	Photocrosslinking	Extrusion	penicillin–streptomycin	HDFs (1 × 10^6^ mL^−1^)			Increased cytocompatibility Support of HDF attachment and proliferation Promising marine-based biomaterial for skin repair
QSHs [161]	EDC-NHS coupling			NIH-3T3			Enhanced short and long-term biocompatibility/cell viability
CELLINK Skin [139]	Submersion in a thrombin-based solution	Pneumatic bioprinting		HDFa and HEKa	Fibrinogen scaffold		Cell viability and growth

GelMAl: gelatin methacryloyl; CUR: curcumin; ADSCs: adipose-derived stem cells; rhCol3: type III re-combinant human collagen; HDFs: human dermal fibroblasts; HaCaTs: human epidermal keratinocytes; SD: Sprague-Dawley; GelMAm: gelatin-methacrylamide; HAMA: methacrylated hyaluronic acid; PCL: polycaprolactone; HUVECs: human umbilical vein endothelial cells; NHDFs: normal human dermal fibroblasts; GAH: gelatin–sodium alginate hydrogels; ADSCs: adipose-derived mesenchymal stem cells; GSH: macroporous scaffolds with gradient stiffness; SA: sodium alginate; HMVECs: human dermal microvascular endothelial cells; NHEKs: normal human epidermal keratinocytes; SF: silk fibroin; GMA: glycidyl methacrylate; DLP: digital light processing; EGF: epidermal growth factor; TE: tissue engineering; GH/AT: hydroxy-phenyl propionic acid-conjugated gelatin and tyramine-modified alginate; NIH3T3: mouse fibroblast cells; hUC-MSCs: human umbilical cord mesenchymal stem cells; PU: polyurethane; EPCs: endothelial progenitor cells; DM: diabetes mellitus; PF127-SH: thiolated pluronic F127; dECM: decellularized extracellular matrix; GelMA: gelatin methacrylate: hADSCs: human adipose-derived stem cells; PRP: platelet-rich plasma; AG: alginate–gelatin; DFs: dermal fibroblasts; ESCs: epidermal stem cells; ECM: extracellular matrix; FSA: functionalized sodium alginate; DNA: deoxyribonucleic acid; CEpCUR: curcumin-loaded particles of cellulose esters; DCEL: diethylaminoethyl cellulose; MC: methyl cellulose; *S. aureus*: *Staphylococcus aureus*; *P. aeruginosa*: *Pseudomonas aeruginosa*; pDAM2: porcine decellularized adipose matrix; XG: xanthan gum; GTA: glutaraldehyde; CMCh: Carboxymethyl chitosan; HA: hyaluronic acid; NPs: nanoparticles; XRU-MA: methacrylated rhamnose rich xylorham-no-uronic acid extract; QSHs: quince seed hydrocolloids; EDC: 3-(3-Dimethylaminopropyl)-1-ethylcarbodiimide hydrochloride; NHS: N-hydroxysuccinimide; γ-PGA-GMA: glycidyl methacrylate-conjugated γ-polyglutamic acid; γ-PGA-SH: thiolated γ-polyglutamic acid; RGDC: thiolated arginine–glycine– aspartic acid; HUVECvegf165+: vascular endothelial growth factor 165-overexpressed human umbilical vein endothelial cells; HDFa: adult human dermal fibroblasts; HEKa: adult human epidermal keratinocytes.
